# RNF8 promotes efficient DSB repair by inhibiting the pro‐apoptotic activity of p53 through regulating the function of Tip60

**DOI:** 10.1111/cpr.12780

**Published:** 2020-02-07

**Authors:** Hongyu Chen, Jin Shan, Jialing Liu, Yunpeng Feng, Yueshuang Ke, Wenjing Qi, Wenguang Liu, Xianlu Zeng

**Affiliations:** ^1^ The Key Laboratory of Molecular Epigenetics of Ministry of Education Institute of Genetics and Cytology Northeast Normal University Changchun Jilin China; ^2^ Laboratory of Noncoding RNA Institute of Biophysics Chinese Academy of Sciences Beijing China; ^3^ Department of Bioscience Changchun Normal University Changchun Jilin China

**Keywords:** cell apoptosis, cell proliferation, DNA double‐strand break (DSB) repair, p53, RNF8, Tip60

## Abstract

**Objectives:**

RING finger protein 8 (RNF8) is an E3 ligase that plays an essential role in DSB repair. p53 is a well‐established tumour suppressor and cellular gatekeeper of genome stability. This study aimed at investigating the functional correlations between RNF8 and p53 in DSB damage repair.

**Materials and methods:**

In this article, wild‐type, knockout and shRNA‐depleted HCT116 and U2OS cells were stressed, and the roles of RNF8 and p53 were examined. RT‐PCR and Western blot were utilized to investigate the expression of related genes in damaged cells. Cell proliferation, apoptosis and neutral cell comet assays were applied to determine the effects of DSB damage on differently treated cells. DR‐GFP, EJ5‐GFP and LacI‐LacO targeting systems, flow cytometry, mass spectrometry, IP, IF, GST pull‐down assay were used to explore the molecular mechanism of RNF8 and p53 in DSB damage repair.

**Results:**

We found that RNF8 knockdown increased cellular sensitivity to DSB damage and decreased cell proliferation, which was correlated with high expression of the p53 gene. RNF8 improved the efficiency of DSB repair by inhibiting the pro‐apoptotic function of p53. We also found that RNF8 restrains cell apoptosis by inhibiting over‐activation of ATM and subsequently reducing p53 acetylation at K120 through regulating Tip60.

**Conclusions:**

Taken together, these findings suggested that RNF8 promotes efficient DSB repair by inhibiting the pro‐apoptotic activity of p53 through regulating the function of Tip60.

## 1 INTRODUCTION

The human genome is under constant threat from various endogenous and exogenous sources of stress. Double‐strand breaks (DSBs) are one of the most dangerous types of DNA damage because they disrupt chromosome continuity.^1^, ^2^ Failure to eliminate DSBs leads to genome instability and tumorigenesis.^1^, ^3^ DSBs are repaired by two major systems: non‐homologous end joining (NHEJ) and homologous recombination (HR). NHEJ is an intrinsically error‐prone repair system that operates throughout the cell cycle. HR is an error‐free repair system limited to the late S and G2 phases because sister chromatids are present at only these times.^4^, ^5^, ^6^

Upon DSB formation, the following processes occur: the histone H2AX is phosphorylated by ataxia telangiectasia mutated (ATM), and mediator of DNA damage checkpoint 1 (MDC1) binds to phosphorylated H2AX (γ‐H2AX). E3 ubiquitin ligase RING finger protein 8 (RNF8) binds to phosphorylated MDC1 at DSB sites and promotes the recruitment of RNF168, another E3 ubiquitin ligase. RNF8 and RNF168 conjugate Lys63‐linked ubiquitin chains onto histone H2A with their cognate E2 conjugating enzyme UBC13 to induce chromatin remodelling.^7^, ^8^, ^9^, ^10^ RNF8/RNF168‐UBC13‐dependent ubiquitination of histones at DSBs promotes the assembly of a series of repair proteins, including BRCA1 and 53BP1. Thus far, RNF8 has been shown to promote DSB damage repair mainly by promoting the binding of key proteins involved in DSB damage repair to DSB sites through RNF8 ubiquitination activity.^9^, ^11^, ^12^, ^13^, ^14^ In addition to the classic role of RNF8 described above, other effects of RNF8 have been reported. Studies have confirmed that RNF8 depletion partially suppresses HR defects caused by BRCA1 depletion, and the degree of HR suppression due to RNF8 depletion is significantly less than that due to RNF168 depletion.^15^ Moreover, scientists revealed a newly discovered non‐catalytic role of RNF8 in unfolding higher‐order chromatin structures.^16^ Furthermore, RNF8 has been shown to promote cellular resistance to replication stress and is important for meiosis.^17^, ^18^, ^19^, ^20^, ^21^ Thus, RNF8 may maintain genomic stability through multiple pathways.

Different levels of damage may trigger different responses; low levels of DNA damage trigger repair and cell survival, and high levels of DNA damage trigger cell death.^22^ p53 is a well‐established tumour suppressor and cellular gatekeeper of genome stability.^23^ p53 stabilization may contribute to the different expression levels of pro‐survival and pro‐apoptosis genes.^24^, ^25^ p53 has been shown to determine cell fate through promoting cell survival or apoptosis in many works, but the specific mechanism of this role remains to be revealed. RNF8 and p53 are both key repair factors in the DSB damage response. In this report, we found that the expression of p53 was increased in RNF8‐deficient cells compared to that in control cells and that increased p53 was highly correlated with the increased sensitivity of RNF8‐deficient cells to DSB damage. Although RNF8 and p53 are present in the same complex, RNF8, an E3 ubiquitin ligase, neither degrades nor ubiquitinates p53. However, RNF8 can effectively improve the efficiency of DSB damage repair through weakening p53 acetylation at the K120 site by regulating Tip60 activity. The present results indicate that RNF8 promotes efficient DSB repair by inhibiting the pro‐apoptotic function of the p53 gene and reveals a novel way by which RNF8 regulates DSB damage repair efficiency.

## 2 MATERIALS AND METHODS

### 2.1 Antibody and reagents

Anti‐p53 (sc‐126), anti‐RNF8 (sc‐271462), anti‐BRCA1(sc‐6954) and anti‐53BP1(sc‐22760) were obtained from Santa Cruz Biotechnology (Dallas, TX); anti‐MDM2 (D155246‐0025), anti‐BAX(Ab‐167) and anti‐p21 (D120403) were obtained from BBI Life Sciences (shanghai, China); anti‐β actin (66009‐1‐Ig), anti‐MCM6 (13347‐2‐AP) and anti‐ATM (27156‐1‐AP) were obtained from Proteintech (Shanghai, China); anti‐γ‐H2AX (Ser319) (#9718S) was obtained from Cell Signaling Technology (Boston, MA); anti‐γ‐H2AX (Ser319)( 05‐636) and anti‐ATM(ser1981)(10H11.E12) were obtained from Calbiochem^®^ Minipore (Darmstadt,Germany); anti‐Flag (F1804) and anti‐HA (H9658) were obtained from Sigma‐Aldrich (Merck KGaA Darmstadt, Germany); anti‐GST (HT601) and anti‐GFP (HT801) were obtained from TransGen Biotech (Beijing, China); anti‐p53(120ac)(YK0089) was obtained from ImmunoWay (Texas‐Plano, USA); anti‐H4K8ac (BS74016) and anti‐K4K16ac (BS94076) were obtained from Bioworld Technology (Nanjing, China). Etoposide(Eto), camptothecin(CPT), 4‐hydroxytamoxifen (4‐OHT) (dissolved in dimethyl sulfoxide (DMSO), ≤ 0.1%), puromycin (dissolved in DMEM) and Duolink^®^ In Situ Detection Reagents Red (DUO92008) were all purchased from Sigma‐Aldrich (Merck KGaA Darmstadt, Germany); propidium iodide (PI) solution was purchased from DingGuo (Beijing, China); GFP‐Trap^®^ _A was purchased from Chromotek (Shanghai, China); DMEM and RPMI 1640 medium were obtained from Gibco (Shanghai, China); TRIzol reagent, PrimeScriptTM RT reagent Kit and SYBR^®^ Premix Ex TaqTM were all purchased from TaKaRa (Kusatsu, shiga, Japan); Lipofectamine 2000 reagent was purchased from Invitrogen (Shanghai, China); Annexin V‐FITC (AO2001‐02) and Annexin V‐APC (AO2001‐11) Apoptosis Analysis Kits were obtained from Sungene Biotech (Tianjin, China). Cell counting kit (CCK‐8) was obtained from San‐bang (Changchun, China). ECL Plus Western blot detection reagents were obtained from US Everbright Inc (San Ramon, CA).

### 2.2 Cell culture and treatment

The A03_1 cell line carrying an array of LacO operators were kindly given by Dr Guohong Li (Institute of Biophysics, Chinese Academy of Sciences, China).^26^ Human HCT116^WT^ and HCT116^p53−/−^ cells were kindly given by Dr Yong Cai (Jilin University, China).^27^ DR‐GFP‐U2OS cells were kindly given by Dr Yungui Yang (Beijing Institute of Genomics, Chinese Academy of Sciences, China). EJ5‐GFP‐U2OS cells were a gift from Dr Xingzhi Xu (Capital Normal University, China). U2OS cells (American Type Culture Collection), U2OS‐shCTR cells, U2OS‐shRNF8 cells, MCF7 cells (American Type Culture Collection), MCF7‐shCTR cells, MCF7‐shRNF8 cells, DR‐GFP‐U2OS cells, EJ5‐GFP‐U2OS cells, Asi‐ER‐U2OS cells and 293T cells (American Type Culture Collection) were all cultured in DMEM supplemented with 10% foetal bovine serum (FBS); HCT116 ^WT^‐shCTR cells, HCT116 ^WT^‐shRNF8 cells, HCT116^p53−/−^‐shRNF8 cells were all cultured in RPMI 1640 supplemented with 10% foetal bovine serum (FBS); A03_1 cells were cultured in F‐12 Ham's medium (Gibco) supplemented with 10% foetal bovine serum (FBS) (Gibco) and 1% penicillin/streptomycin (Invitrogen). All cells above were cultivated at 37°C and 5% CO_2_ in a humidified incubator. For DSB induction, cells were incubated with the indicated concentrations of Eto or CPT for 20 min and then released for repair. AsiSI‐ER‐U2OS cells were treated with 300 nM 4‐OHT for 3 h to induce DSBs.

### 2.3 Plasmids

pmCherry‐LacI vector (for LacI‐LacO targeting) plasmid was generously provided by Dr Guohong Li and Dr Ruiming Xu; the full‐length cDNA of human Tip60, DYRK2 and p53 was cloned into a pmCherry‐LacI vector with restriction enzyme site of EcoR I (upstream) and Kpn I (downstream); pcDNA3.1(−/−)‐Flag vector, pcDNA3.1(−/−)‐Flag‐p53 and pcDNA3.1(−/−)‐Flag‐Tip60 plasmids were kindly provided by Dr Yong Cai (Jilin University, China); the full‐length cDNA of human DYRK2 was cloned into a pcDNA3.1(−/−)‐Flag vector with restriction enzyme site of Xho I (upstream) and Kpn I (downstream); HA‐RNF8 plasmid was generously provided by Dr Shinichiro Nakada^28^; GFP‐RNF8 plasmid was kindly provided by Dr Nico P Dantuma^16^; Myc‐MDM2 plasmid was kindly provided by Dr Xiaomeng Li (Northeast Normal University, China); RFP‐I‐scel plasmid was kindly provided by Dr Xingzhi Xu (Capital Normal University, China); the pBABE HA‐ER‐AsiSI plasmid was generously provided by Gaelle Legube; p‐Cherry, NHEJ‐GFP and DR‐GFP plasmids were kindly provided by Dr Huadong Pei (Beijing Institute of Radiation Medicine, China); GST‐p53 plasmid was constructed by cloning full‐length cDNA of p53 into a pGEX‐4T‐2 vector (Addgene). To stably knock down RNF8 in U2OS cells, MCF7 cells and HCT116 cells, the RNF8 shRNA and shCTR sequences were synthesized and cloned into the pLKO.1—TRC Cloning Vector (Addgene); PLKO.1‐EGFP‐Puro‐shNC, PLKO.1‐EGFP‐Puro‐shTip60#1, PLKO.1‐EGFP‐Puro‐shTip60#2 and PLKO.1‐EGFP‐Puro‐shTip60#3 plasmids were all purchased from Fenghui Biotechnologies Inc (Hunan, China). In addition, PEGFP‐N1 vector, psPAX2 and pMD2.G plasmids were purchased from Addgene. All primers and shRNA for plasmids construction are present in Table 1 and Table 2.

**Table 1:** Primers used for constructing expression plasmids

Name	Forward primer (5′‐3′)	Reverse primer (5′‐3′)
pmCherry‐LacI‐p53	CGGAATTCTGCCACCATGGAGGAGCCGCAGTCAGAT	GGGGTACCTCAGTCTGAGTCAGGCCCTTC
pmCherry‐LacI‐Tip60	CGGAATTCTGCCACCATGGCGGAGGTGGGGGAGATA	GGGGTACCTCACCACTTCCCCCTCTTGCT
pmCherry‐LacI‐DYRK2	CGGAATTCTGCCACCATGAATGATCACCTGCATGTC	GGGGTACCTCAGCTAACAAGTTTTGGCAA
Flag‐DYRK2	CCCTCGAGGCCACCATGAATGATCACCTGCATGTC	GGGGTACCTCAGCTAACAAGTTTTGGCAA
GST‐p53	CGGAATTCCCATGGAGGAGCCGCAGTCAGAT	CCCTCGAGTCAGTCTGAGTCAGGCCCTTC

**Table 2:** Sequence used for constructing shRNA plasmids

Name	Sequence
TRC‐PLKO.1‐shCTR	ACGCTGAGTACTTCGAAATGT
TRC‐PLKO.1‐shRNF8	CCAAAGAATGACCAAATGATA
PLKO.1‐EGFP‐Puro‐shNC	GTTCTCCGAACGTGTCACGT
PLKO.1‐EGFP‐Puro‐shTip60#1	GGACGTAAGAACAAGAGTTAT
PLKO.1‐EGFP‐Puro‐shTip60#2	CCTCAATCTCATCAACTACTA
PLKO.1‐EGFP‐Puro‐shTip60#3	TCGAATTGTTTGGGCACTGAT

### 2.4 Plasmid transfection and lentiviral infection

Most of the transfection experiments were performed according to the manufacturer's instructions of Lipofectamine™ 2000. For pBABE HA‐ER‐AsiSI plasmid, briefly, the transfections were performed in 60 mm dishes containing 70% confluent cells with 4 µg DNA per dish. AsiSI‐ER‐U2OS cells stably transfected with the pBABE HA‐AsiSI‐ER plasmid were selected using 1 mg/mL puromycin. Isolated individual Asi‐U2OS clones were further validated by Western blotting. For lentiviral transduction, the TRC‐PLKO.1‐shRNF8, TRC‐PLKO.1‐shCTR, PLKO.1‐EGFP‐Puro‐shNC, PLKO.1‐EGFP‐Puro‐shTip60#1, PLKO.1‐EGFP‐Puro‐shTip60#2 and PLKO.1‐EGFP‐Puro‐shTip60#3 plasmids were transfected into HEK‐293T cells separately, together with the packaging plasmid psPAX2 and the envelope plasmid pMD2.G by using the Lipofectamine 2000 reagent. The lentiviral supernatants were harvested at 48, 72, 96 h after transfection; then, the virus was concentrated and injected the cells. The infected cells were selected in puromycin‐containing medium for 3 days after infection.

### 2.5 Homologous recombination (HR) and non‐homologous end joining (NHEJ) repair analysis

In gene overexpression analysis, HR and NHEJ were, respectively, measured in DR‐GFP‐U2OS and EJ5‐GFP‐U2OS cells according to previous publications.^29^, ^30^ DR‐GFP‐U2OS cells and EJ5‐GFP‐U2OS cells (1 × 10^5^ per well) grown in six‐well plates were pre‐transfected with indicated plasmids. Twenty‐four hours later, cells were transfected with the RFP‐I‐SceI expression plasmid (2 μg/well). Forty‐eight hours later, cells were harvested and analysed. In gene knockdown analysis, HCT116^WT^, HCT116^WT^‐shCTR, HCT116^p53−/−^ and HCT116^p53−/−^‐shRNF8 cells were used. For HR pathway, the DR‐GFP and RFP‐I‐SceI plasmids (1:1) were transfected into different kinds of HCT116 cells, and the repair efficiency of HR was detected 48 h after transfection. For NHEJ pathway, the NHEJ‐GFP plasmid needs to be first digested by *Hind* III enzyme for linearization; then, the linearized NHEJ‐GFP and p‐Cherry plasmids (3:1) were transfected into different kinds of HCT116 cells, and the repair efficiency of NHEJ was detected 36 h after transfection. All cells were harvested and analysed for RFP‐positive cells and RFP/GFP both positive cells by flow cytometry. For each analysis, 1 × 10^4^ cells were collected, and each experiment was repeated three times. We then divided the number of RFP/GFP both positive cells with RFP single‐positive cells to get the relative percentage of GFP‐positive cells.

### 2.6 Protein expression and GST pull‐down assay

Escherichia coli strain BL‐21 (DE3) was transformed with indicated plasmids and cultured overnight. GST fusion protein expression was induced with IPTG (isopropyl β‐D‐thiogalactoside). Cells were harvested in lysis buffer (20 mmol/L Hepes (pH 7.5), 120 mmol/L NaCl, 10% glycerol, 2 mmol/L EDTA, 1 mg/mL lysozyme, 1 mmol/L PMSF, 10 μg/mL each aprotinin and leupeptin) and homogenized by sonication. After centrifugation, GST fusion proteins in supernatant were purified by glutathione Sepharose 4B bead according to the manufacturer's instructions (Amersham Pharmacia Biotech). For GST pull‐down assay, HEK‐293T cells were lysed with RIPA (Radio Immunoprecipitation Assay) lysis buffer (50 mmol/L Tris‐HCl, pH 7.4, 150 mmol/L NaCl, 1% Triton X‐100, 1% sodium deoxycholate, 1% SDS, 1 mmol/L EDTA, 1 mmol/L Na3VO4, 2 mmol/L NaF, 1 mmol/L β‐glycerophosphate and 2.5 mmol/L sodium pyrophosphate, 1 mmol/L PMSF and protease inhibitors). Cell lysates were incubated with 10 μL beads coated with GST or GST‐p53 fusion proteins for 3 hours. The beads were collected by centrifugation and washed with ice‐cold lysis buffer. After boiling in Laemmli sample buffer, the coimmunoprecipitated proteins were detected by immunoblotting.

### 2.7 Microscopic imaging

For immunofluorescence (IF), cells grown on glass coverslips were fixed with 10% (w/v) formaldehyde in PBS for 10 min and then permeabilized with 0.5% (v/v) Triton X‐100 for 5 min. After permeabilization, cells were washed and blocked in 10% FBS for 30 min. The cells were incubated with the primary antibody, washed and stained with a secondary antibody. For laser microirradiation, U2OS cells were grown on coverslips and incubated in Hoechst 33 342 (2 μg/mL) for 5 min. Then, cells were irradiated with pulsed nitrogen laser (50 Hz, 405 nm) at 85% output power for 10 s, prior to fixation by ice‐cold methanol on ice for 10 min, and cells were pre‐extracted in buffer D (10 mmol/L PIPES pH 7.0, 100 mmol/L NaCl, 300 mmol/L sucrose, 3 mmol/L MgCl_2_, 0.5% Triton X‐100) to exclude the soluble non‐chromatin binding proteins. Cells were then washed and blocked as described above, then stained with indicated antibodies. For LacI‐LacO targeting system staining, A03_1 cells grown on glass coverslips were transfected with indicated plasmids for 48 hours, then fixed with 10% (w/v) formaldehyde in PBS for 10 minutes and stained with DAPI. For proximity ligation assay (PLA), U2OS cells grown on glass coverslips were transfected with indicated plasmids for 48 hours and then fixed with 4% (w/v) paraformaldehyde for 15 minutes. The PLA was performed using the Duolink^®^ In Situ Detection Reagents Red (DUO092008) from Sigma‐Aldrich following the manufacturer's guidance. All images were taken using confocal microscope (FluoView FV1000, Olympus).

### 2.8 Neutral cell comet assay

The neutral comet assay was performed using the Comet Assay Kit from Trevigen (Gaithersburg, MD) following the manufacturer's guidance. Images were captured using the fluorescent microscope (ECLIPSE, 80i, Nikon, Japan). Tail moment was tested using CometScore software (TriTek, Sumerduck, USA).

### 2.9 Immunoprecipitation (IP) and Western blotting

The cells were lysed with RIPA lysis buffer, and the whole cell lysates were incubated with appropriate antibodies at 4°C for 3 hours. Samples were then incubated for another 3 hours with protein A/G Sepharose beads. After washing the samples with RIPA lysis buffer, the immunoprecipitates were resolved on SDS‐PAGE and probed with antibodies as indicated.

### 2.10 Flow cytometry

γ‐H2AX was monitored in U2OS cells after Eto treatment, incubation for the indicated time intervals and fixation in 70% ethanol overnight. Cells were permeabilized (0.25% Triton X‐100 on ice for 15 min), washed, incubated overnight in PBS containing 0.1% BSA and γ‐H2AX antibodies, and then incubated with the secondary antibody for 30 minutes at room temperature. Cells were incubated in 50 mg/mL propidium iodide and 100 mg/mL RNase A for 30 minutes, and 1 × 10^4^ cells per sample were analysed. For apoptosis assay, Annexin V‐FITC and Annexin V‐APC Apoptosis Analysis Kits were used according to the manufacturer's instructions. Briefly, cells were harvested, washed with cold PBS, suspended in 1 × binding buffer and centrifuged at 300 *g* for 10 min. The supernatant was removed, and the cell pellet was resuspended in 100 μL of binding buffer with 5 μL of Annexin V‐FITC and incubated at room temperature for 10 minutes. After a gentle vortex, 5 µL of PI solution was added, and the mixture was incubated for another 5 minutes at room temperature, and 1 × 10^4^ cells per sample were analysed. For cell cycle analysis, U2OS‐shCTR and U2OS‐shRNF8 cells were washed with PBS, detached by adding trypsin and collected by centrifugation at 1000 rpm for 5 minutes. Then, the cells were washed with cold PBS and fixed in 70% ethanol for 24 hours. The cells were then washed twice with cold PBS, resuspended in PBS solution with 50 µg/mL RNase A and incubated for 30 minutes at 37°C. The suspension was then added to propidium iodide (PI) solution (10 µg/mL) and incubated in the dark for 15 minutes. The stained cells were analysed for relative DNA content by flow cytometry. Flow cytometry was performed using a FACSCanto II (BD Biosciences, San Jose, CA), and the data were analysed with FACSDiva software (BD Biosciences).

### 2.11 Mass spectrometry

After staining protein in SDS‐PAGE gels with coomassie blue, gel lanes were sliced into different bands. The specific analysis process was completed by Shanghai Applied protein Technology (APT), China.

### 2.12 Quantitative real‐time polymerase chain reaction (qRT‐PCR)

Total RNA was isolated from cultured cells using the TRIzol reagent, and the resulting messenger RNA (mRNA) was reversely transcribed into complementary DNA (cDNA) using PrimeScript^™^ RT reagent Kit according to the instructions. QRT‐PCR analysis was performed using SYBR^®^ Premix Ex Taq^™^ following the introductions. GAPDH, 18SrRNA and β‐actin were all served as the internal reference. The method of 2^−ΔΔCt^ was used to calculate the relative mRNA expression of the target genes. According to the mRNA sequences of target genes and reference genes in GenBank, the primers were all designed by Primer Premier 5 (Table 3).

**Table 3:** Primers used for qRT‐PCR analyses

Name	Forward primer (5′‐3′)	Reverse primer (5′‐3′)
p21^a^	CCCGTGAGCGATGGAACT	CGAGGCACAAGGGTACAAGA
BAX^b^	ACCAAGAAGCTGAGCGAGTGT	ACAAACATGGTCACGGTCTGC
PUMA^c^	GCTGCCCGCTGCCTACCTCT	GGCCCACTGTTCCTCCTCCC
p53AIP1^d^	GTGATGCCTCCGAATGGC	CAACCTCAACGGTGCTTT
p53^e^	GTTTCCGTCTGGGCTTCT	ACCTCAGGCGGCTCATAG
β‐Actin	CTCCATCCTGGCCTCGCTGT	GCTGTCACCTTCACCGTTCC
18s rRNA^f^	CAGCCACCCGAGATTGAGCA	TAGTAGCGACGGGCGGTGTG
GAPDH^g^	AACGGATTTGGTCGTATTG	GGAAGATGGTGATGGGATT
Tip60^h^	TGGAATACCGTCAGCACC	TGGAAGCCCTTACAGTCATAC

a p21, cyclin‐dependent kinase inhibitor 1A.

b BAX, Bcl‐2‐associated X, apoptosis regulator.

c PUMA, BCL2 binding component 3.

d p53AIP1, tumour protein p53‐regulated apoptosis‐inducing protein 1.

e p53, tumour protein p53.

f 18s rRNA, 18S ribosomal RNA.

g GAPDH, glyceraldehyde‐3‐phosphate dehydrogenase.

h Tip60, lysine acetyltransferase 5.

### 2.13 Cell proliferation assay

Different types of cells were first treated with the indicated concentrations of Eto or DMSO for 20 minutes and then trypsinized, counted and plated in dishes. For CCK‐8 assay, 100 μL of cell suspension was seeded on 96‐well dishes at a density of 3 × 10^3^/well, and with 0, 1, 2, 3 and 4 day as time points of observation. Then, 10 μL of CCK‐8 reagent was added into each well, and the OD value was measured 4 hours later at wavelength of 450 nm with a microplate reader. For clone formation assay, the suspended cells were seeded on 12‐well dishes (5000 cells/dish). Following a 4‐day recovery period, the cells were fixed in 50% (vol/vol) methanol/0.01% (wt/vol) crystal violet solution for 5 minutes and washed three times with water (10 min/wash). The number of proliferating cells was determined based on the staining density of crystal violet according to the ImageJ software.

### 2.14 Statistical analysis

The data were analysed using the Student t test and presented as the mean ± SEM. The quantifications were based on the results of at least three independent experiments. The levels of significance are designated *P < .05, **P < .01 and ***P < .001.

## 3 RESULTS

### 3.1 RNF8 knockdown increases the sensitivity of cells to DSBs

**Figure 1:**
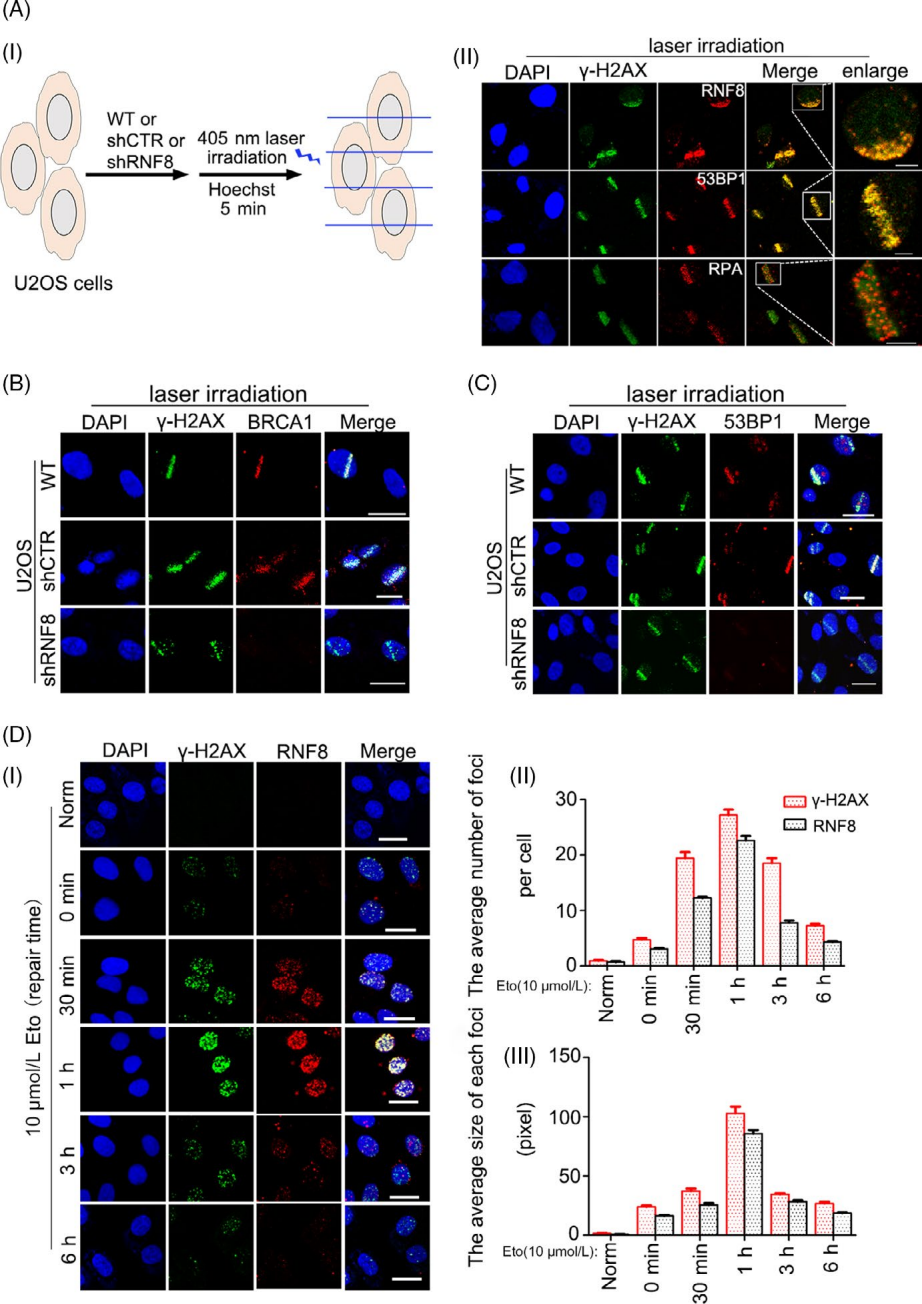
RNF8 participates in multiple types of DSBs damage response. A, (i) A schematic diagram showing the use of laser microirradiation. (ii) U2OS cells were laser microirradiated and allowed to repair for 1 h. The cells were then fixed and immunostained with anti‐γ‐H2AX, anti‐RNF8, anti‐53BP1 and anti‐RPA antibodies. Scale bars = 10 μm. B and C, U2OS cells stably transfected with shCTR or shRNF8 plasmids were laser microirradiated and allowed to repair for 1 h. The cells were then fixed and immunostained with anti‐γ‐H2AX, anti‐BRCA1 and anti‐53BP1 antibodies. Scale bars = 20 μm. D, (i) Endogenous γ‐H2AX and RNF8 in U2OS cells were detected by immunofluorescence. Cells were treated with 10 μmol/L Eto for 20 min, then repaired for the indicated time intervals and stained with DAPI, anti‐γ‐H2AX and anti‐RNF8 antibodies. Scale bars = 20 μm. (ii) Quantification of the number of γ‐H2AX and RNF8 foci per cell. (iii) Quantification of the average size of each foci

In response to DSB damage, RNF8 provides a structural chromatin platform for the subsequent recruitment of key DNA repair factors, such as 53BP1 and BRCA1. 9, 14, 31 To confirm the function of RNF8 in DSB damage repair, we detected the role of RNF8 in different types of DSB damage. RNF8 was enriched at laser‐induced DSB sites, which were marked by γ‐H2AX. Similar to 53BP1 and γ‐H2AX, RNF8 was associated with chromatin, which is different from RPA, a single‐stranded binding protein (Figure 1A).^12^, ^32^, ^33^ RNF8 deficiency impeded the recruitment of BRCA1 and 53BP1 at DSB sites (Figure 1B, C and Figure S1A). Restrictive endonuclease can also induce DNA splicing and cause endogenous DSB damage. U2OS cells stably expressing the HA‐tagged AsiSI‐ER restriction enzyme were employed to generate fragments of the human genome longer than 1 Mb.^34^ RNF8 was also recruited at restriction enzyme‐induced DSB sites and co‐localized with γ‐H2AX (Figure S1B‐D). Then, the drug etoposide (Eto), a topoisomerase II inhibitor, was used to induce DSB damage. Eto interferes with topoisomerase II activity and causes DSBs through the formation of a DNA‐drug‐enzyme cleavage complex.^35^ RNF8 was recruited to Eto‐induced DSBs. Trends in the average number and size of RNF8 foci in each damaged cell were similar to those of γ‐H2AX, the number of which peaked after 1 hour of repair (Figure 1D). The above results indicate that RNF8 is a chromatin‐associated protein widely involved in the repair of damage from DSBs from different sources, including laser irradiation, restriction enzymes and chemical drugs, and which is consistent with the classical way by which RNF8 regulates DSB damage repair efficiency.

**Figure 2:**
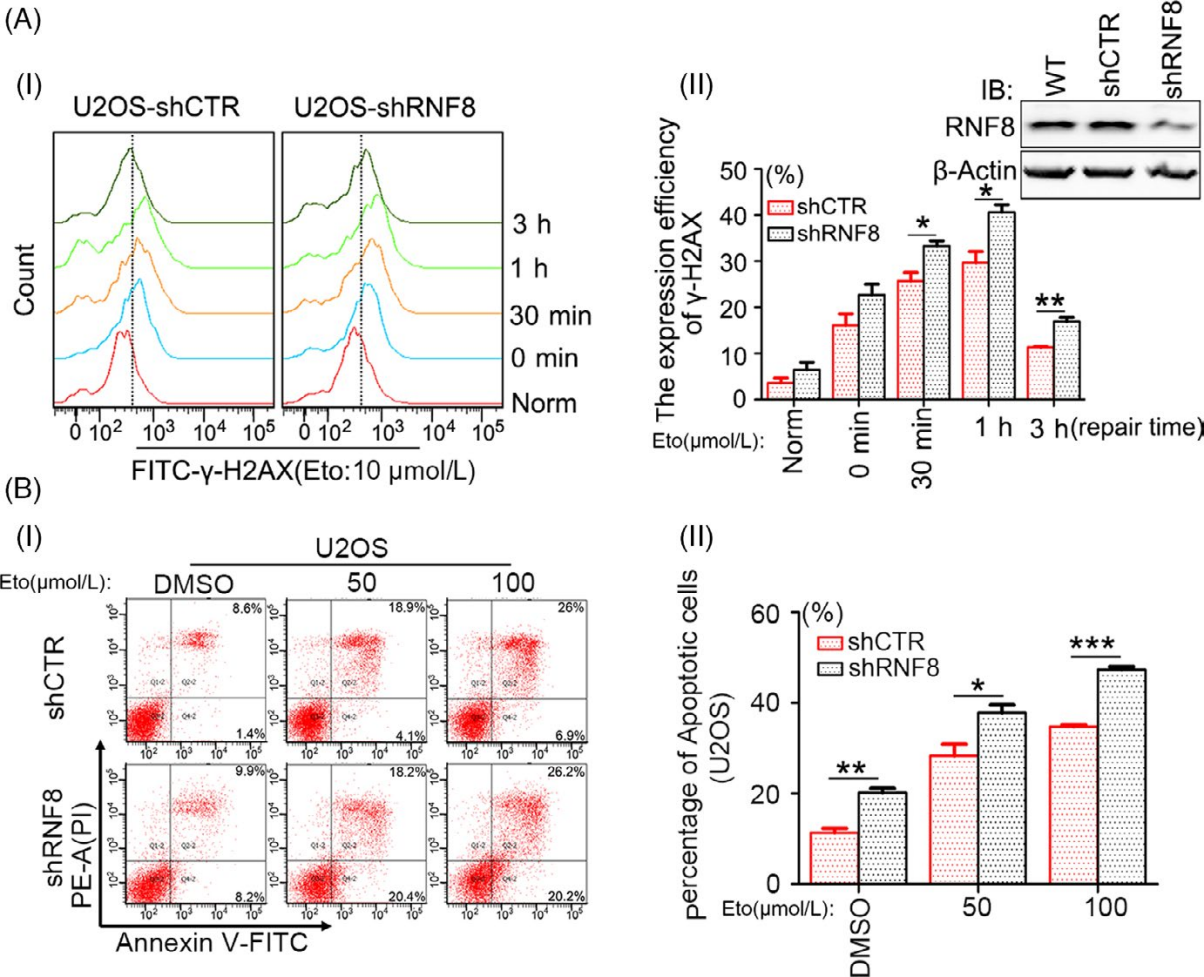
Knock down of RNF8 increases the sensitivity of cells to DSBs. A, (i) U2OS cells stably transfected with shCTR or shRNF8 plasmids were treated with 10 μmol/L Eto for 20 min and allowed to repair for the indicated time intervals. Flow cytometric analysis of γ‐H2AX staining. 1 × 10^4^ cells were analysed in a single experiment, and the data shown are from a single representative experiment out of three replicates. (ii) Quantification of γ‐H2AX‐positive cells. *P < .05, **P < .01, n ≥ 3. The interference efficiency of cells was detected by Western blot. B‐(i) The differently treated U2OS cells were incubated with 0, 50, or 100 μmol/L of Eto for 24 h, and flow cytometry analysis of Annexin V‐FITC/PI staining was conducted to examine the dead cells. (ii) The total number of Annexin V‐positive and Annexin V/PI double‐positive cells is quantified. *P < .05, **P < .01, ***P < .001, n ≥ 3

To explore the function of RNF8 in DNA damage, the effect of RNF8 expression on DSB repair efficiency following Eto treatment was examined. RNF8‐proficient and RNF8‐deficient U2OS cells were established, and changes in the formation of γ‐H2AX foci following Eto treatment were examined. Compared with Eto‐treated control cells, U2OS‐shRNF8 cells contained more γ‐H2AX foci, and more time was needed for the signal from these foci to decrease to the background level (Figure 2A), suggesting reduced repair efficiency. In addition, RNF8 knockdown significantly increased cell apoptosis compared with that in U2OS‐shCTR cells (Figure 2B). The above results indicate that RNF8 knockdown increases the sensitivity of cells to DSB damage.

### 3.2 The increased sensitivity of RNF8‐deficient cells to DSB damage is correlated with high expression levels of the p53 gene

**Figure 3:**
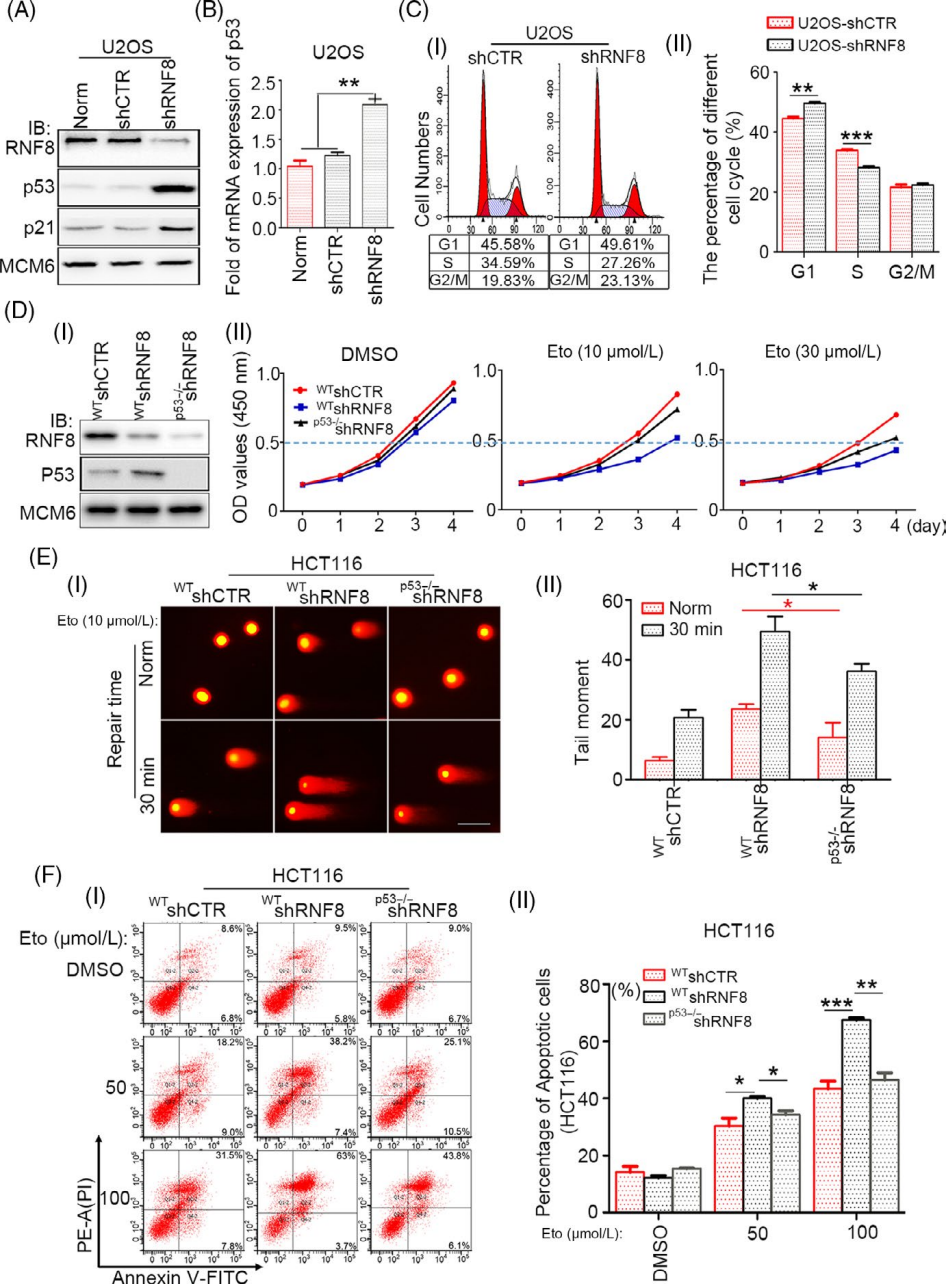
The increased sensitivity of RNF8‐deficient cells to DSBs is correlated with the high expression of p53 gene. A, Western blot analysis of the depletion efficiency of RNF8 and the expression efficiency of p53 and p21 in differently treated U2OS cells, and MCM6 was used as loading control. B, Real‐time PCR analysis of the expression efficiency of p53 in differently treated U2OS cells. C, (i). Flow cytometry analysis of cell cycle distribution of U2OS‐shCTR and U2OS‐shRNF8 cells. (ii) The percentage of cells in each cell cycle phase. **P < .01, ***P < .001, n ≥ 3. D‐(i) Western blot analysis of the depletion efficiency of RNF8 and p53 in differently treated HCT116 cells. (ii) CCK‐8 experiment shows that the proliferation ability of differently treated cells. HCT116^WT^‐shCTR, HCT116^WT^‐shRNF8 and HCT116^p53−/−^‐shRNF8 cells were treated with DMSO, 10 or 30 μmol/L Eto for 20 min and then replated. The observations were made at 0, 1, 2, 3, 4 days. E, (i) DSBs in differently treated HCT116 cells exposed to 10 μmol/L Eto and repaired for 30 min were analysed by Neutral comet assay. Scale bar = 30 μm. (ii) The olive tail moment was determined by using CometScore software, and more than 50 individual comets were counted for each sample. F‐(i) The different types of HCT116 cells were incubated with 0, 50, or 100 μmol/L of Eto for 24 h, and flow cytometry analysis of Annexin V‐FITC/PI staining was conducted to examine the dead cells. (ii) The total number of Annexin V‐positive and Annexin V/PI double‐positive cells is quantified. *P < .05, **P < .01, ***P < .001, n ≥ 3

Since cell apoptosis is correlated with the expression and activation of p53,^36^, ^37^, ^38^ the protein and mRNA levels of p53 were first examined. p53 at both the protein and mRNA levels was increased in shRNA‐transfected U2OS cells (Figure 3A, B) and also in MCF7 cells treated the same way (Figure S2A, B). Many studies have shown that p53 can promote G1/S cell cycle arrest, 39 so we examined the cell cycle distribution of U2OS‐shCTR and U2OS‐shRNF8 cells. It was found that compared with cells with normal expression of RNF8, the proportion of cells with low expression of RNF8 increased in G1 phase and decreased significantly in S phase (Figure 3C). To further explore whether the increased cell sensitivity caused by RNF8 knockdown is related to increased p53 expression, HCT116^p53−/−^ cells were developed, and the effects of RNF8 and p53 on the sensitivity of HCT116 cells to Eto were examined. Stable HCT116^WT^‐shCTR, HCT116^WT^‐shRNF8 and HCT116^p53−/−^‐shRNF8 cells were established and treated with different concentrations of Eto, and the proliferation of HCT116^WT^‐shRNF8 cells was slower than that of HCT116^WT^‐shCTR cells. However, the proliferation of HCT116^p53−/−^‐shRNF8 cells was faster than that of HCT116^WT^‐shRNF8 cells (Figure 3D, Figure S3A, B). Then, a neutral comet assay was conducted, and the degree of DNA breaks in HCT116^p53−/−^‐shRNF8 cells was significantly lower than that in HCT116^WT^‐shRNF8 cells (Figure 3E). Furthermore, the proportion of apoptotic HCT116^p53−/−^‐shRNF8 cells was much lower than that of apoptotic HCT116^WT^‐shRNF8 cells (Figure 3F). The above results indicate that the increased sensitivity of RNF8‐deficient cells to DSB damage is closely related to the high‐level expression of the p53 gene.

### 3.3 RNF8 can improve DSB repair efficiency by inhibiting the pro‐apoptotic function of p53

**Figure 4:**
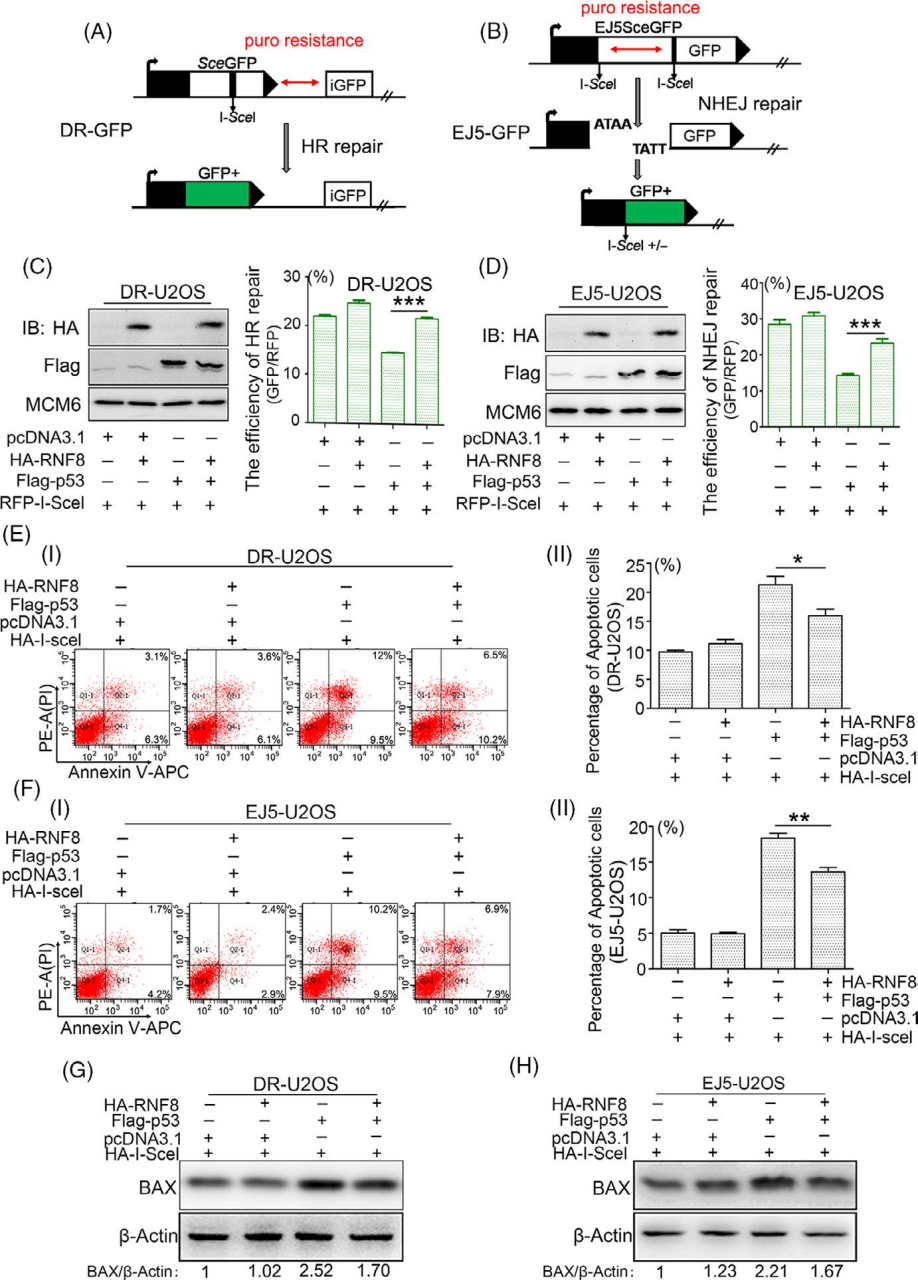
RNF8 improves the repair efficiency of DSBs by inhibiting the pro‐apoptotic function of p53. A, Schematic of the DR‐GFP reporter used to monitor homologous recombination (HR) repair. puro, puromycin resistance gene. B, Schematic of the EJ5‐GFP reporter used to monitor non‐homologous end joining (NHEJ) in U2OS cells. C and D, DR‐GFP‐U2OS and EJ5‐GFP‐U2OS cells were transfected with indicated plasmids. After 72 h, RFP‐positive cells and GFP/RFP‐double‐positive cells were analysed by flow cytometry, and the repair efficiency of HR and NHEJ were shown by the ratio of <GFP : RFP>. The Western blot results showed the expression of the HA‐RNF8 and Flag‐p53 proteins. E‐(i) and F‐(i) DR‐U2OS or EJ5‐U2OS cells were transfected with indicated plasmids for 72 h, and flow cytometry analysis of Annexin V‐APC/PI staining was conducted to examine the dead cells. E, (ii) and F‐(ii) The total number of Annexin V‐positive and Annexin V/PI double‐positive cells was quantified. *P < .05, **P < .01, n ≥ 3. G and H. Western blot analysis of the expression of BAX in differently treated cells. β‐Actin was used as the loading control

Based on the above results, we speculated that RNF8 improves the efficiency of DSB damage repair, promotes cell survival and maintains genomic stability by inhibiting the functional activity of p53. To verify the effect of RNF8 expression on the two DSB damage repair pathways, HR and NHEJ, two well‐characterized GFP‐based reporter systems, DR‐GFP and EJ5‐GFP, were utilized under conditions of p53 activation. The system was described previously.^29^, ^30^ A schematic diagram is presented in Figure 4A, B, and the cells were treated as shown in Figure S4. Overexpression of p53 mimics the sustained activation of p53 in damaged cells. p53 overexpression significantly inhibited the effects of both HR and NHEJ repair pathways. However, when RNF8 and p53 were both overexpressed, the efficiencies of the HR and NHEJ repair pathways were both significantly improved (Figure 4C, D). In the gene knockdown assay on HCT116 cells, we found that the repair efficiency of HR and NHEJ was reduced in cells with low expression of RNF8, while the efficiency of HR and NHEJ repair was improved in cells with low expression of RNF8 and p53 (Figure S5A, B). In addition, the number of apoptotic DR‐GFP and EJ5‐GFP U2OS cells expressing high levels of RNF8 and p53 was significantly lower than that of DR‐GFP and EJ5‐GFP U2OS cells expressing only p53 (Figure 4E, F). In addition, in both DR‐GFP and EJ5‐GFP U2OS cells, the overexpression of p53 alone promoted an increase in the apoptosis‐related protein Bcl‐2‐associated X protein (BAX), which is the downstream target protein of p53; however, the simultaneous overexpression of p53 and RNF8 reduced the level of BAX (Figure 4G, H and Figure S6A, B). Therefore, our collective data suggest that RNF8 promotes DSB damage repair by inhibiting the pro‐apoptotic function of p53.

### 3.4 RNF8 indirectly regulates the pro‐apoptotic function of p53

**Figure 5:**
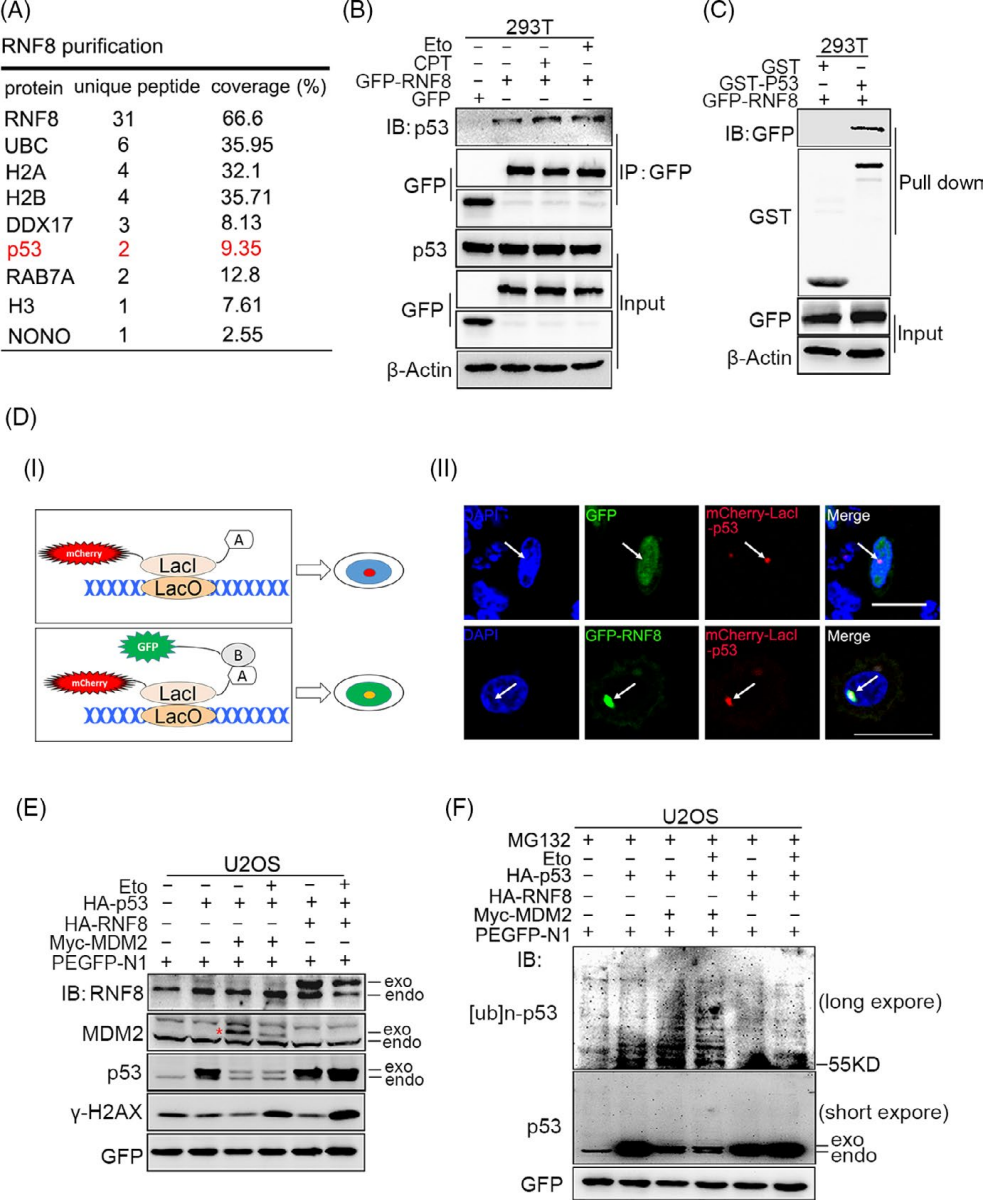
RNF8 indirectly regulates the pro‐apoptotic function of p53. A, Co‐immunoprecipitation (Co‐IP) purification experiment was performed using HEK‐293T cells expressing GFP‐tagged RNF8 as indicated in the method. The part of hits from mass spectrometry analysis was shown. B, Co‐IP assays were performed to check the interaction between RNF8 and p53 in Eto or CPT treated HEK‐293T cells. IP samples were resolved by SDS‐PAGE and immunoblotted with the indicated antibodies. C, GST pull‐down assay of p53 was conducted using the indicated proteins expressed in bacteria. GFP‐tagged RNF8 was expressed in HEK‐293T cells, and the lysates were incubated with purified GST or GST‐fused p53. Bound proteins were separated by SDS‐PAGE and immunoblotted with anti‐GFP antibody. D, (i) A schematic diagram showing the lac operator‐repressor (LacO‐LacI) targeting system for assaying co‐localization and interaction of proteins. (ii) Co‐localization of p53 with GFP and GFP‐RNF8. E, U2OS cells were transfected with indicated plasmids for 48 h, then treated with or without Eto (10 μmol/L). The immunoblot showed the expression of the indicated proteins. GFP was used as the loading control. F, The cells were treated as E and exposed to 10 mmol/L MG132 (a potent cell‐permeable 20S proteasome inhibitor) for 6 h before protein lysate extraction. GFP was used as the loading control

The results above suggest that RNF8 can inhibit the pro‐apoptotic activity of p53 during DSB damage repair. To explore the exact mechanism of this inhibitory effect, we first tested whether p53 and RNF8 are present in the same complex during DSB damage repair. GFP‐tagged RNF8 was expressed in HEK‐293T cells, and RNF8 complexes were then isolated and subjected to mass spectrometry. A number of known RNF8‐associated proteins (UBC,^40^ H3^41^ and NONO^42^) were co‐purified with RNF8, and p53 was also present in the RNF8 complex (Figure 5A). In addition, GST pull‐down and immunoprecipitation assays confirmed the presence of RNF8 and p53 in the same complex (Figure 5B, C). Furthermore, by using a lac operator‐repressor (LacO‐LacI) targeting system, we found that p53 indeed co‐localized with RNF8 (Figure 5D), suggesting a functional correlation between p53 and RNF8. RNF8 is an E3 ubiquitin ligase that ubiquitinates many substrates and promotes the degradation of substrates such as H3 and NONO.^42^, ^43^ However, in our experimental system, RNF8 could neither degrade p53 nor promote its ubiquitination (Figure 5E, F and Figure S7). Therefore, although RNF8 and p53 are present in the same complex, RNF8 does not seem to have a direct regulatory effect on p53.

### 3.5 RNF8 inhibits the pro‐apoptotic function of p53 by regulating the activity of Tip60

**Figure 6:**
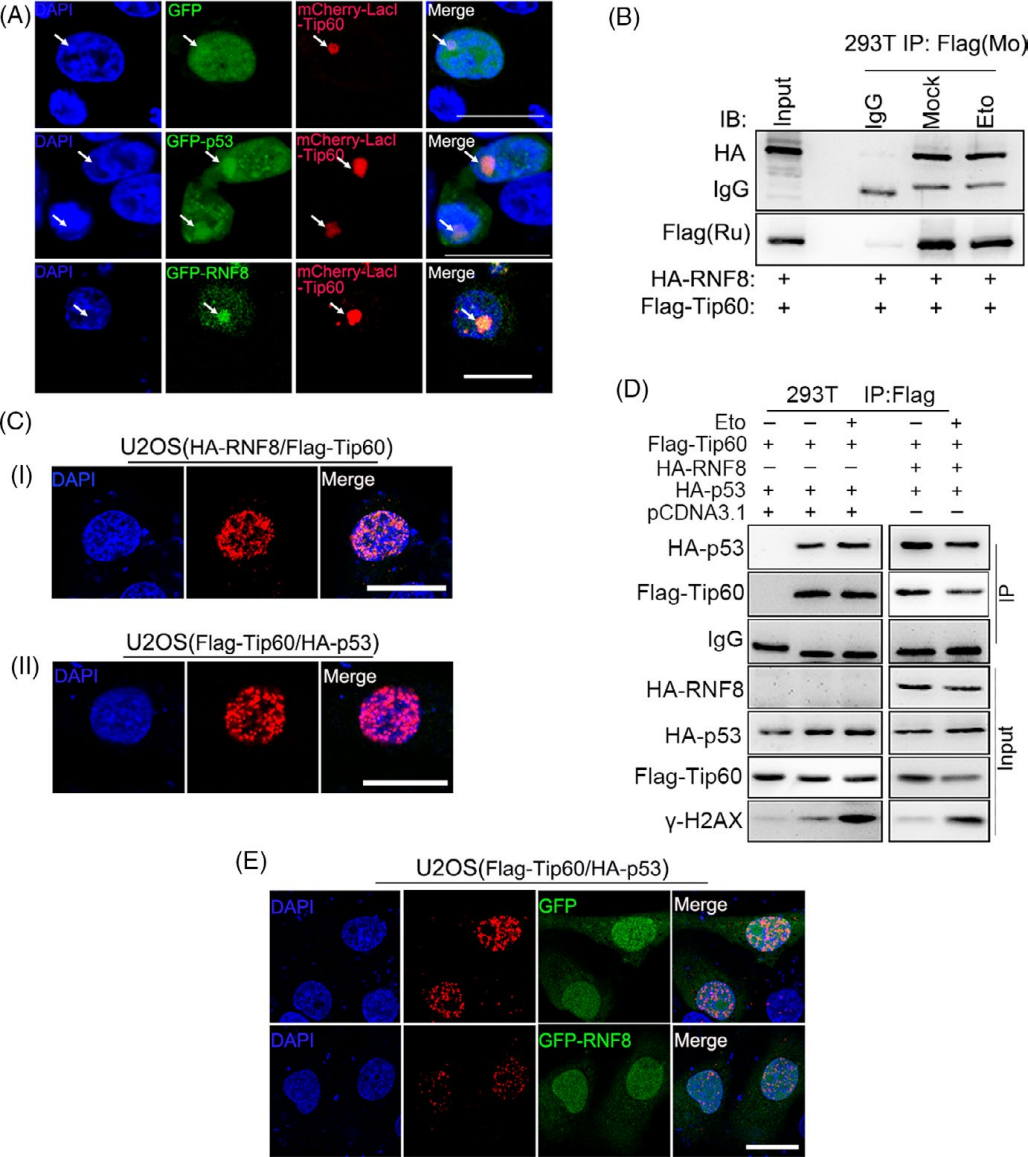
RNF8 inhibits the pro‐apoptotic function of p53 by regulating the functions of Tip60. A, Co‐localization of Tip60 with GFP, GFP‐p53 and GFP‐RNF8. B, Co‐IP assays were performed using HEK‐293T cells to check the interaction between RNF8 and Tip60 with or without Eto treatment. Co‐IP sample was resolved by SDS‐PAGE and immunoblotted with the indicated antibodies. C, (i), (ii) The indicated plasmids were transfected into U2OS cells, and in situ PLA using proximity probes against HA and Flag was performed to visualize HA‐RNF8/Flag‐Tip60 heterodimers and Flag‐Tip60/HA‐p53 heterodimers in cultured human U2OS cells. Scale bars = 20 μm. D, Co‐IP assays were performed using HEK‐293T cells to check the interaction between Tip60 and p53. The indicated plasmids were transfected into HEK‐293T cells, and then, the cells were treated with or without Eto 36 h later. E. The cells were treated as that in C. Scale bars = 20 μm

**Figure 7:**
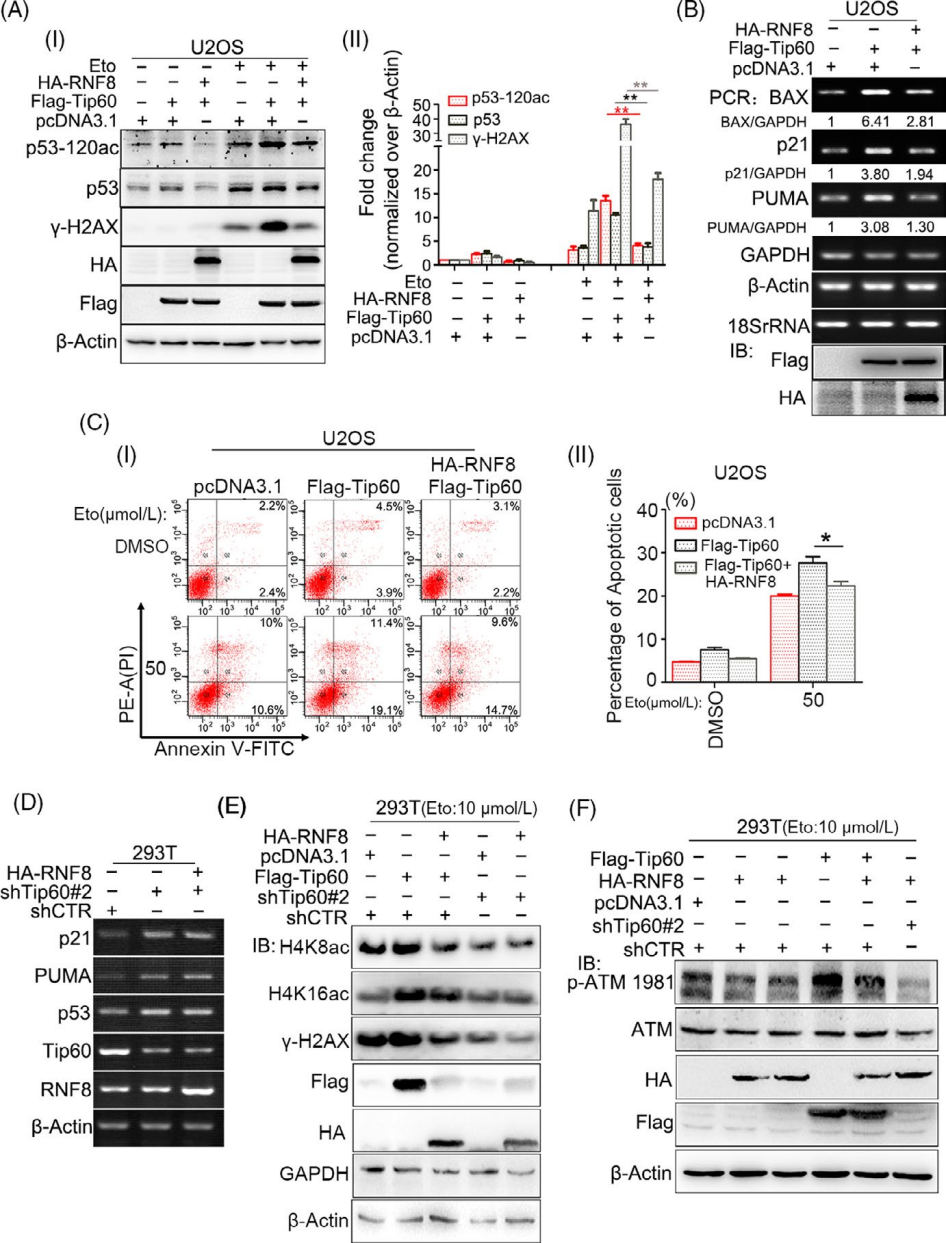
RNF8 can inhibit p53‐dependent apoptosis by attenuating Tip60‐dependent excessive activation of ATM. A, (i). U2OS cells were transfected with indicated plasmids for 48 h, then treated with or without Eto (10 μmol/L). The expression of the indicated proteins was showed by immunoblot. β‐Actin was used as the loading control. (ii) The quantification of p53‐120ac, p53 and γ‐H2AX protein levels. **P < .01, n ≥ 3. B, U2OS cells were transfected with indicated plasmids for 48 h, and total RNA was extracted and reversely transcribed into cDNA, and the expression of related genes was detected by PCR. C, (i) U2OS cells were transfected with indicated plasmids for 48 h and then incubated with 0, 50 μmol/L of Eto for 24 h, and the flow cytometry analysis of Annexin V‐FITC/PI staining was conducted to examine the dead cells. (ii) The total number of Annexin V‐positive and Annexin V/PI double‐positive cells was quantified. *P < .05, n ≥ 3. D, Different types of HEK‐293T cells were transfected with indicated plasmids for 48 h, and total RNA was extracted and reversely transcribed into cDNA, and the expression of related genes was detected by PCR. E and F, Different types of HEK‐293T cells were transfected with indicated plasmids for 48 h; then, the cells were treated with Eto (10 μmol/L) for 20 min and repaired 1 h. The immunoblot showed the expression of the indicated proteins. β‐Actin and GAPDH were used as the loading control

Based on the results above, we preliminarily speculated that the inhibition of the pro‐apoptotic function of p53 by RNF8 is indirectly mediated by other proteins. The pro‐apoptotic activity of p53 depends on its acetylation at K120 by the acetyltransferase Tip60.^44^ So we first checked whether RNF8 can regulate the function of Tip60. Tip60 co‐localized with not only p53 but also RNF8 (Figure 6A). Protein immunoprecipitation experiments showed that RNF8 and Tip60 are present in the same complex under normal or DNA damage conditions (Figure 6B). In order to detect whether there is direct interaction between RNF8 and Tip60, we conducted the proximity ligation assay (PLA). The results showed that both endogenously expressed (Figure S8A) and exogenously expressed (Figure 6C i) RNF8 and Tip60 were directly interacted. In addition, a direct interaction between Tip60 and p53 was also detected (Figure 6C ii and Figure S8B). We also found that the interaction between Tip60 and p53 was attenuated both in immunoprecipitation assay (Figure 6D) and PLA (Figure 6E) when RNF8 was overexpressed. Furthermore, RNF8 significantly inhibited the Tip60‐mediated acetylation of p53 at K120, and when RNF8 and Tip60 were simultaneously overexpressed, p53 acetylation at K120 (Figure 7A and Figure S9A) and the expression of p53‐induced downstream apoptosis‐related genes, such as BAX, p21 and PUMA (Figure 7B and Figure S9B), were significantly decreased, compared to that in cells overexpressing Tip60 alone. Consistent with the above results, the apoptosis rate in cells overexpressing RNF8 and Tip60 together was significantly lower than that in cells overexpressing Tip60 alone (Figure 7C).

Therefore, RNF8 can directly attenuate the interaction between Tip60 and p53.

To further investigate whether the regulation of RNF8 on the pro‐apoptotic function of p53 is dependent on the presence of Tip60, we stably interfered Tip60 (Figure S10A) and found that RNF8 does not affect the expression of apoptosis‐related target genes downstream of p53 (Figure 7D) and the death of cells (Figure S10B) with low expression of Tip60. In order to further explore the mechanism of RNF8 in regulating Tip60, we identified whether RNF8 affects the acetyltransferase activity of Tip60. The results showed that overexpression of Tip60 can significantly enhance the acetylation levels of H4K8 and H4K16, while the acetylation levels of H4K8 and H4K16 are significantly down‐regulated in the cells co‐expressing RNF8 and Tip60 and the cells with low expression of Tip60, and the co‐expression of RNF8 and Tip60 also significantly decreased the expression of γ‐H2AX compared with the cells expressing Tip60 alone (Figure 7E). Tip60 can form complexes with ATM and acetylate ATM to promote the auto‐phosphorylation and activation of ATM.^45^ Our results also showed that high expression of Tip60 can significantly enhance the phosphorylation modification of ATM‐ser1981 site. Compared with the cells expressing Tip60 alone, the phosphorylation level of ATM‐ser1981 is lower in the cells with low expression of Tip60 or expressing of Tip60 and RNF8 at the same time (Figure 7F). The above results indicate that RNF8 can inhibit p53‐dependent apoptosis by Tip60‐dependent excessive activation of ATM.

## 4 DISCUSSION

Upon DNA damage, cells respond to stress in ways ranging from the activation of survival pathways (eg, DNA repair and the activation of cell cycle checkpoints) to the initiation of cell death (eg, cell senescence and apoptosis) that eventually eliminate damaged cells.^46^, ^47^ Cells enter a protective or destructive stress response depending on the nature and duration of the stress as well as the cell type. Additionally, there is often interplay among these responses that ultimately determines the fate of the stressed cells.^47^, ^48^ RNF8 is an E3 ubiquitin ligase localized primarily in the nucleus during interphase. In contrast, under genotoxic stress, RNF8 is localized to sites of DNA damage, where it ubiquitinates many chromatin substrates, including histones H2A, H2AX and H1, the ubiquitination of which is of great importance to chromatin structure remodelling.^12^, ^49^ In addition to its classic features above, after DSB damage, RNF8 can also promote efficient DSB damage repair by decreasing the pro‐apoptosis activity of p53 through regulating Tip60 protein activity.

We found that RNF8 is widely involved in the repair of DSB damage induced by multiple sources, such as drugs, restrictive endonucleases and laser irradiation, and foci containing labelled RNF8 overlapped with those containing γ‐H2AX (Figure 1). The loss of RNF8 increased the sensitivity of cells to DSB damage, and p53 was increased at the protein and mRNA levels in RNF8 knockdown cells (Figure 2), indicating that the increased sensitivity of RNF8‐deficient cells to DSB damage is closely related to the high‐level expression of the p53 gene. Marie‐Jo Halaby and colleagues found a synergistic interaction between RNF8 and p53 in the protection against genomic instability and tumorigenesis.^50^ In the present study, we further showed that the proliferation of HCT116^p53−/−^‐shRNF8 cells was faster than that of HCT116^WT^‐shRNF8 cells. In addition, the level of apoptotic HCT116^p53−/−^‐shRNF8 cells was significantly lower than that of HCT116^WT^‐shRNF8 cells (Figure 3). Through the DR‐GFP and EJ5‐GFP repair systems, we also found that RNF8 may promote DSB damage repair by inhibiting the pro‐apoptotic function of p53 (Figure 4).

To further explore the mechanism by which RNF8 inhibits the pro‐apoptotic function of p53, RNF8‐associated complexes were isolated and subjected to mass spectrometry. RNF8 and p53 were present in the same complex; however, RNF8 neither degraded nor ubiquitinated p53 (Figure 5), suggesting that RNF8 indirectly regulates p53 function through mediating other proteins. p53 protein activity in regulating apoptosis is highly correlated with its own post‐translational modification, and the Tip60‐mediated acetylation of p53 at the K120 site is crucial for the activation of PUMA and BAX, which are essential for apoptosis.^51^, ^52^ Indeed, RNF8, p53 and Tip60 were present in the same complex, and the high level of Tip60 expression significantly increased the level of p53 acetylated at the K120 site. However, when RNF8 was overexpressed together with Tip60, the acetylation of p53 at the K120 site was significantly reduced and the expression of apoptosis‐related target genes downstream of p53 (eg, PUMA and BAX) exhibited the same trend (Figure 6 and 7). The results also showed that, compared with the cells expressing Tip60 alone, the protein level of γ‐H2AX in the cells co‐expressing RNF8 and Tip60 was significantly lower. Because the formation of γ‐H2AX is mediated by activated ATM kinase, we suspect that RNF8 may inhibit the ATM activation mediated by Tip60.^45^ To test our hypothesis, we examined the phosphorylation of ATM‐ser1981 at different expression levels of RNF8 and Tip60. We showed that overexpression of Tip60 can significantly enhance the auto‐phosphorylation of ATM‐ser1981; however, the phosphorylation of ATM‐ser1981 is significantly down‐regulated in the cells co‐expressing RNF8 and Tip60 and the cells with low expression of Tip60 (Figure 7). We speculate that RNF8 can inhibit Tip60‐dependent ATM activation. The mechanism of functional regulation of RNF8 for Tip60 is very complex. We suggest that RNF8 may inhibit the p53 pro‐apoptotic function mediated by Tip60 through two pathways. One is the continued expression of γ‐H2AX will activate p53 and promote the apoptosis by inducing the function of p53. RNF8 can inhibit the generation of excessive γ‐H2AX mediated by Tip60. Another is the MDC1 scaffold protein can bind directly to γ‐H2AX, which provide a platform to recruit RNF8 at DSB site. RNF8 can further stabilize the binding of Tip60 to chromatin, which are crucial for the apoptotic response induced by Tip60.^53^ On the contrary, the reduction of γ‐H2AX inhibits the recruitment of RNF8, thus inhibiting the apoptosis‐inducing function of Tip60.

In addition to p53 acetylation, p53 phosphorylation also plays a crucial role in the activation and promotion of apoptosis. The phosphorylation of p53 at Ser46 following severe DNA damage increases the affinity of p53 to the promoters of pro‐apoptotic genes such as p53AIP1.^54^, ^55^ Naoe Taira et al demonstrated that dual‐specificity tyrosine‐phosphorylation‐regulated kinase 2 (DYRK2) can directly phosphorylate p53 at Ser46 upon exposure to genotoxic stress.^56^ In addition, Takenori Yamamoto and colleagues found a direct interaction between DYRK2 and RNF8 in regulating the DNA damage response.^57^ Here, we showed that p53, RNF8 and DYRK2 are co‐localized in the nucleus (Figure S11A); however, we did not detect the direct interaction between RNF8 and DYRK2 reported by Takenori Yamamoto (Figure S11B). This is perhaps due to the different cell types and injury types examined in these studies. In addition, there was no significant difference in the expression of apoptosis‐related genes induced by phosphorylation of p53 at Ser46 under the regulation of RNF8 and DYRK2, such as p53AIP1 and BAX (Figure S11C). Based on the above results, we believe that DYRK2 has a small effect on the regulation of p53 pro‐apoptotic activity by RNF8.

**Figure 8:**
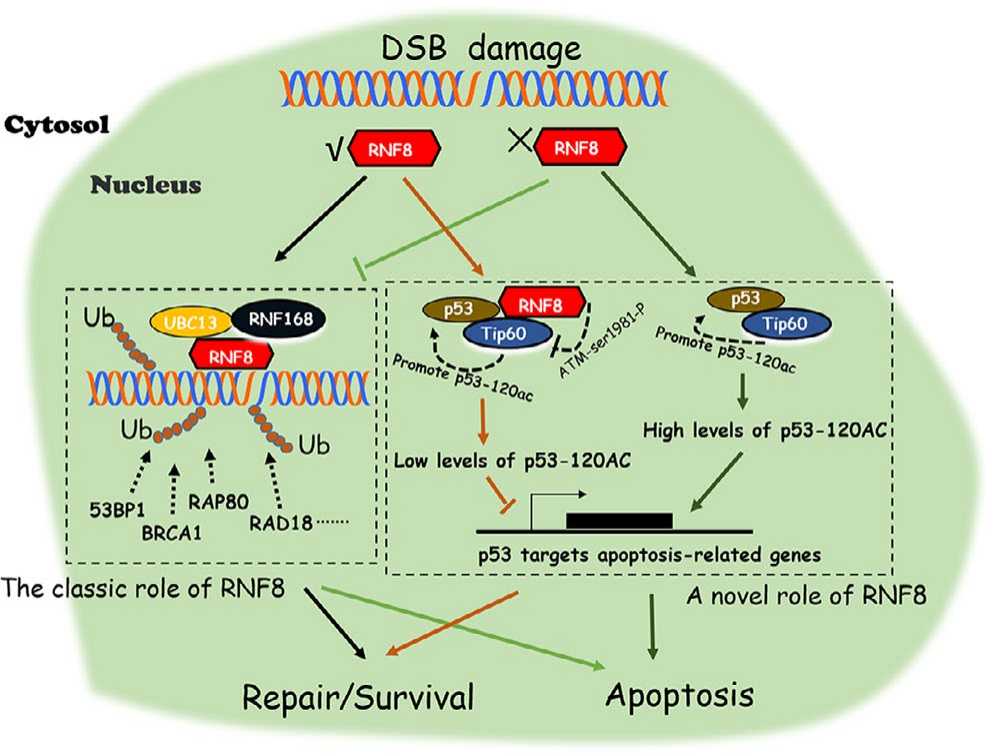
Description of a pattern diagram. Schematic diagram of the possibility of RNF8 in DSBs damage repair. Upon DSBs damage, the expression of RNF8 promotes efficient DSB damage repair by promoting efficient recruitment of repair proteins at DSB sites. In addition to its classic features, RNF8 can also promote DSB damage repair by inhibiting the pro‐apoptotic function of p53 through regulating the acetyltransferase activity of Tip60. RNF8 can reduce the Tip60‐dependent phosphorylation of ATM‐ser1981 and inhibit the over‐activation of ATM kinase. When the expression of RNF8 is low, the acetylation activity of Tip60 cannot be regulated, while ATM kinase will be over‐activated. The synergistic interaction of Tip60 and activated ATM can effectively promote acetylation modification at p53‐K120 site, improve the expression of p53 targeted apoptotic genes and lead to the activation of apoptosis pathway

In summary, our data indicate that RNF8 expression promotes efficient DSB damage repair by not only promoting the efficient recruitment of repair proteins at DSB sites but also inhibiting the pro‐apoptotic function of p53 through attenuating Tip60 activity. RNF8 expression hinders the recruitment of key repair factors at the DSB site. Furthermore, the loss of Tip60 activity regulation effectively promotes the over‐activation of ATM and the acetylation of p53 at K120, which improves the expression of p53‐targeted apoptotic genes and increases apoptosis (Figure 8). Our work not only reveals a new way by which RNF8 regulates the efficiency of DSB damage repair but also provides new details on the effects of p53 on cell fate.

## CONFLICTS OF INTEREST

The authors declare no conflict of interest.

## AUTHOR CONTRIBUTIONS

Study design, XLZ and HYC; methodology, HYC, JS and YPF; software, JS; Data curation, HYC, JS, and JLL; Funding acquisition, XLZ, WGL, YSK, and WJQ; Investigation, HYC, JS and WGL; Validation, XLZ; Writing—original draft, HYC; Writing—review & editing, XLZ and YPF.

## Data Availability

The authors declare that the data supporting the findings of this study are available from the corresponding author upon reasonable request.
