# Supplementary material for: RNF8 promotes efficient DSB repair by inhibiting the pro‐apoptotic activity of p53 through regulating the function of Tip60
Source: Cell Prolif. 2020 Feb 7;53(3):e12780. doi: 10.1111/cpr.12780 (PMC7106964; doi:10.1111/cpr.12780)
Supplement: Supplementary file 1 [file CPR-53-e12780-s001.doc]

**Supporting information**

**Figure S1**


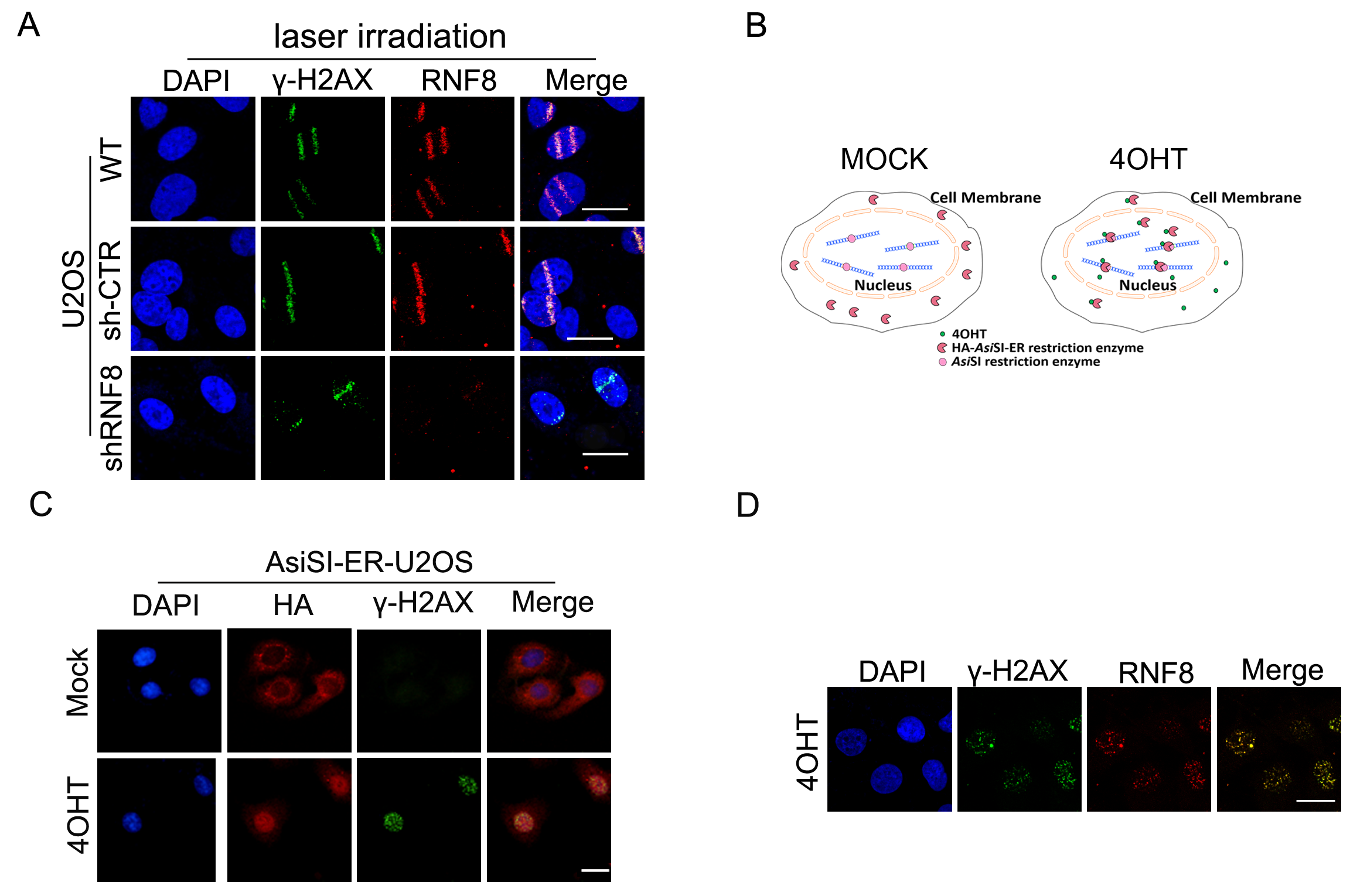


**Figure S1.** RNF8 participate in multiple types of DSBs damage response*.* (**A**) The differently treated U2OS cells were laser microirradiated and allowed to repair for 1 h, and the treated cells were then fixed and immunostained with anti-γ-H2AX and anti-RNF8 antibodies. Scale bars = 20 μm. (**B**) A schematic diagram showing the HA-tagged AsiSI–ER restriction enzyme system for assaying restriction endonuclease induced DNA double strand breaks (DSBs). (**C**) Detection of HA or γ-H2AX by immunofluorescence in Mock and 4-OHT treated AsiSI-ER-U2OS cells. Scale bars = 20 μm. (**D**) Detection of RNF8 or γ-H2AX by immunofluorescence in 4-OHT treated AsiSI-ER-U2OS cells. Scale bars = 20 μm.

**Figure S2**

**
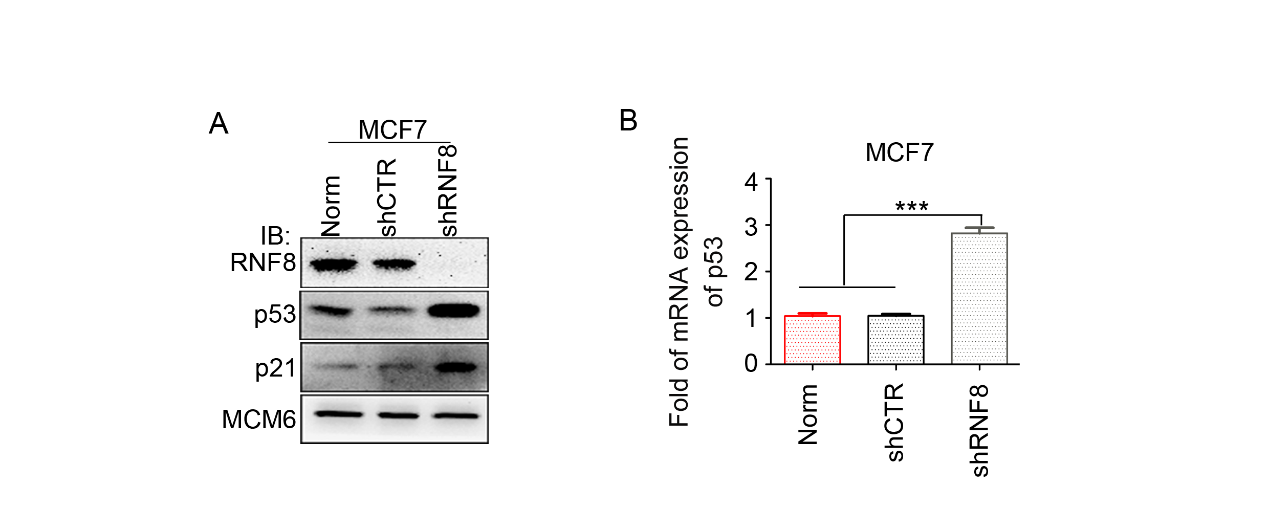
**

**Figure S2.** The increased sensitivity of RNF8-deficient cells to DSBs is correlated with the high expression of p53 gene. (**A**) Western blot analysis of the depletion efficiency of RNF8 and the expression of p53 and p21 in differently treated MCF7 cells. (**B**) Real-time PCR analysis of the expression of p53 in differently treated MCF7 cells.

**Figure S3**

**
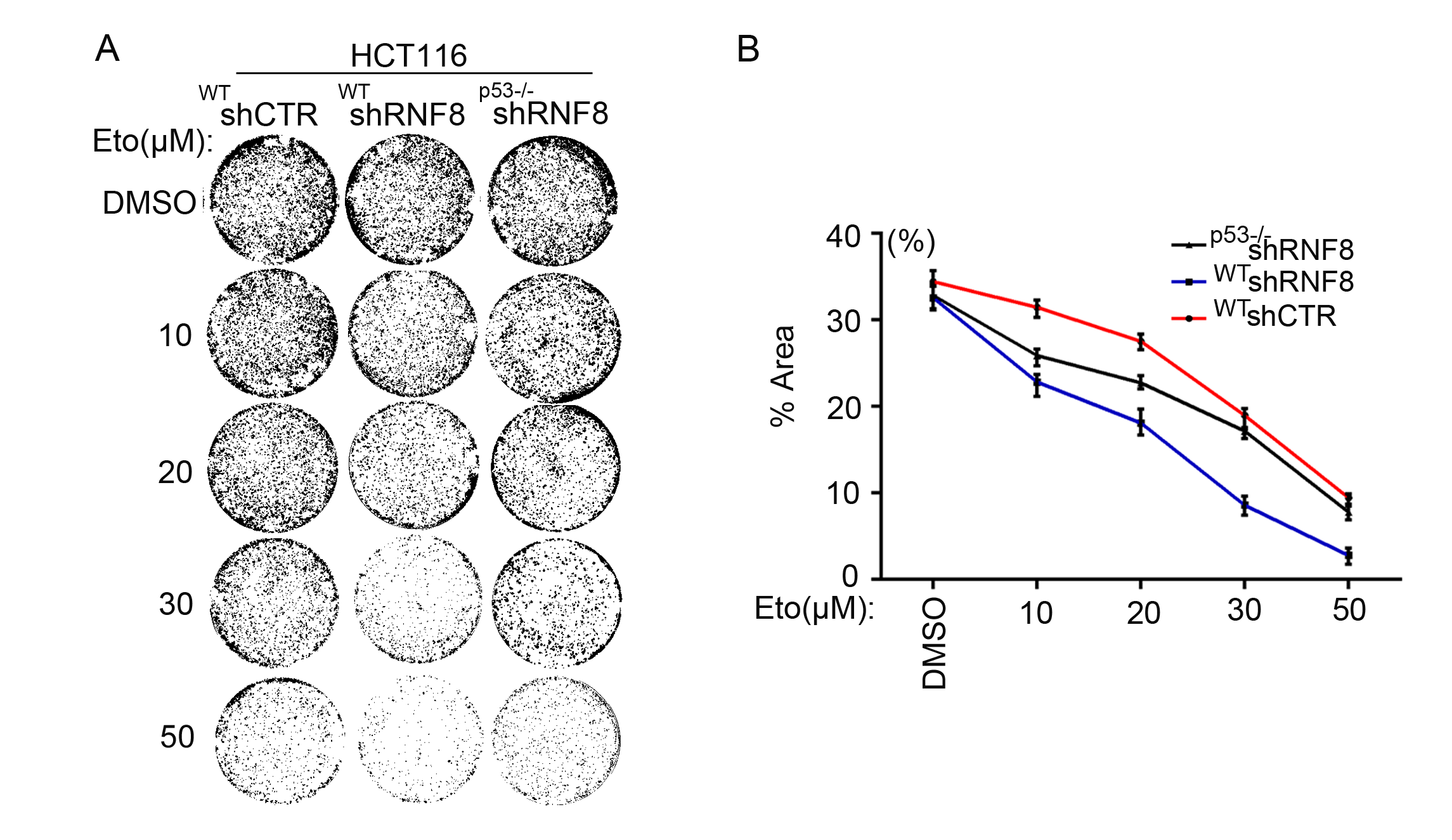
**

**Figure S3.** The proliferation ability of different types of HCT116 cells. (**A**) The indicated cells were treated with 0, 10, 20, 30 or 50 μM Eto for 20 min and then replate. Four days later, the cells were stained with crystal violet. (**B**) The ratio of the dyeing area to the total area was quantified.

**Figure S4**

**
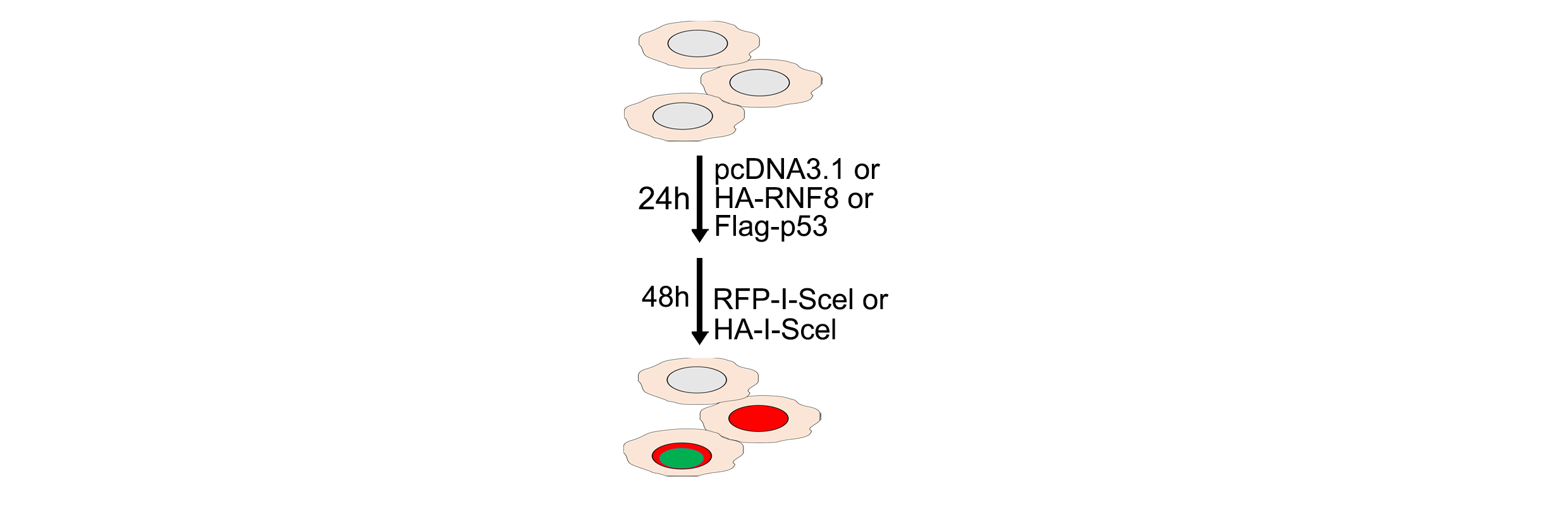
**

**Figure S4.** RNF8 can improve the repair efficiency of DSBs partly by inhibiting the pro-apoptotic function of p53. Schematic diagram of how cells were processed for HR and NHEJ report system.

**Figure S5**

**
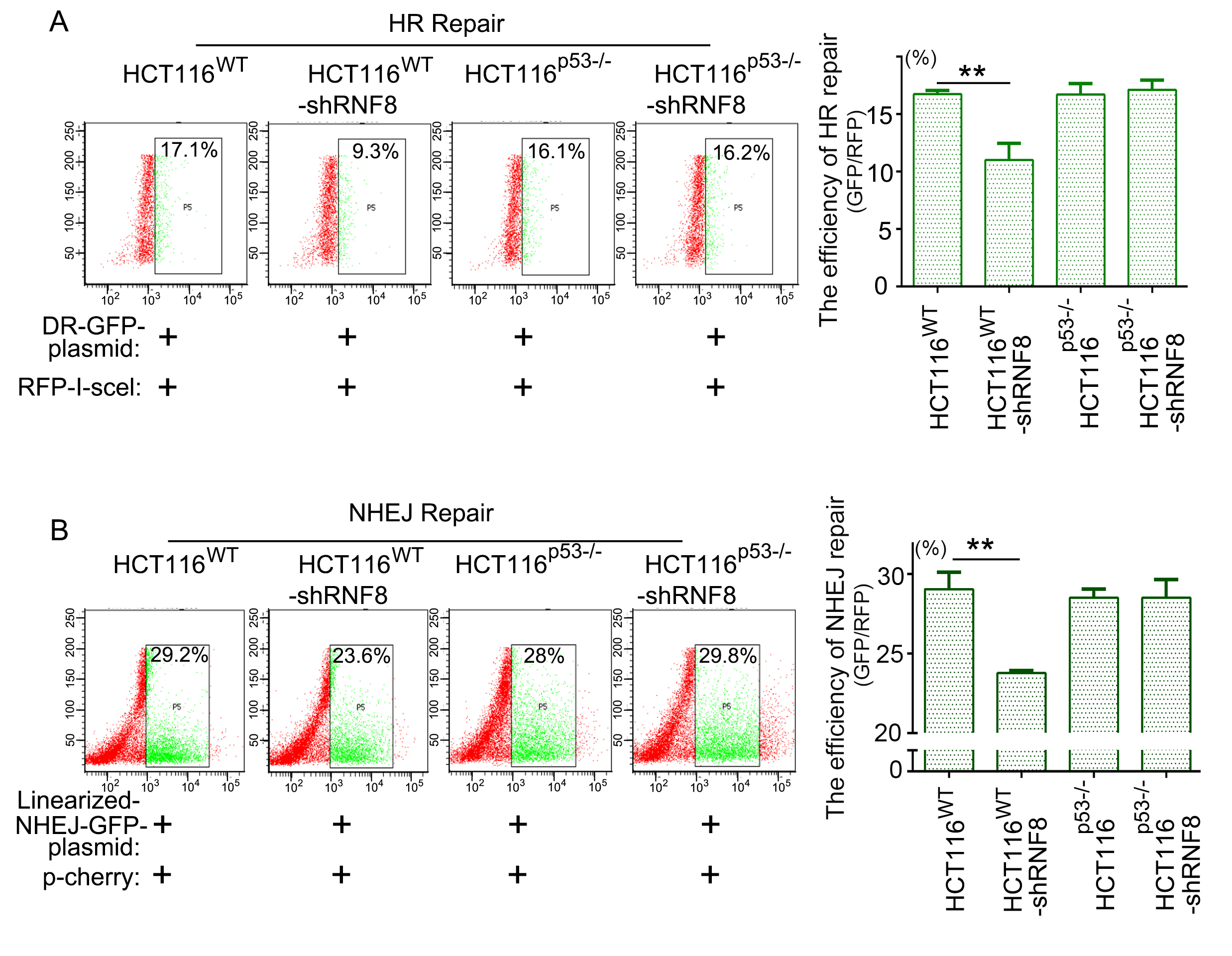
**

**Figure S5**. RNF8 improve the repair efficiency of DSBs by inhibiting the pro-apoptotic function of p53. (A)-(i) and (B)-(i) The different treated HCT116 cells were transfected with indicated plasmids, after 36 h (for NHEJ repair) or 48 h (DR repair), the RFP-positive cells and GFP/RFP-double positive cells were analyzed by flow cytometry. (A)-(ii) and (B)-(ii) the repair efficiency of HR and NHEJ were shown by the ratio of < (GFP: RFP>. **p< 0.01, n≥3.

**Figure S6**

**
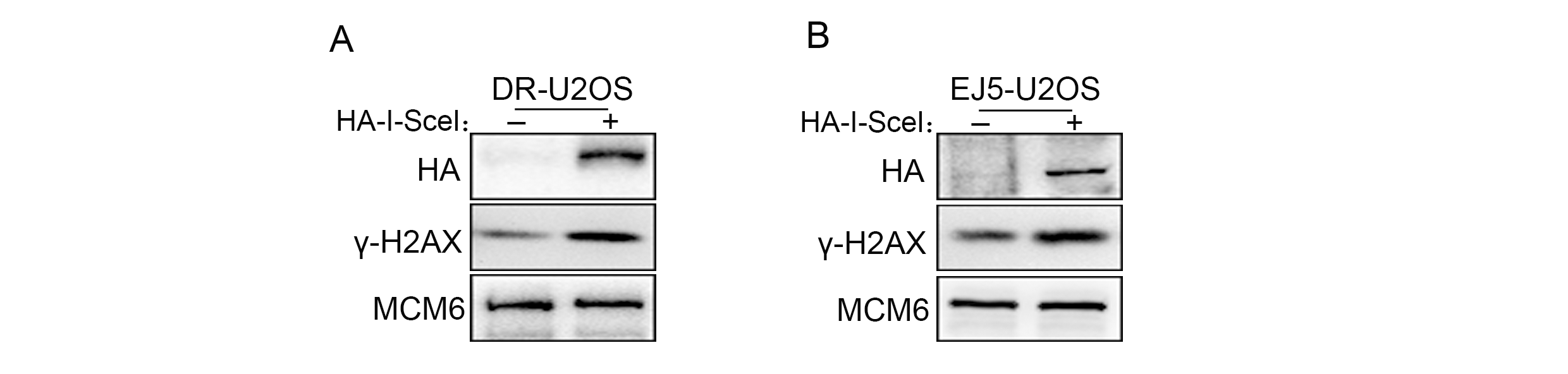
**

**Figure S6.** RNF8 can improve the repair efficiency of DSBs partly by inhibiting the pro-apoptotic function of p53. (A) and (B) Western blot analysis of the transfection and working efficiency of HA-I-Scel in DR-GFP and EJ5-GFP systems, MCM6 was used as loading control.

**Figure S7**

**
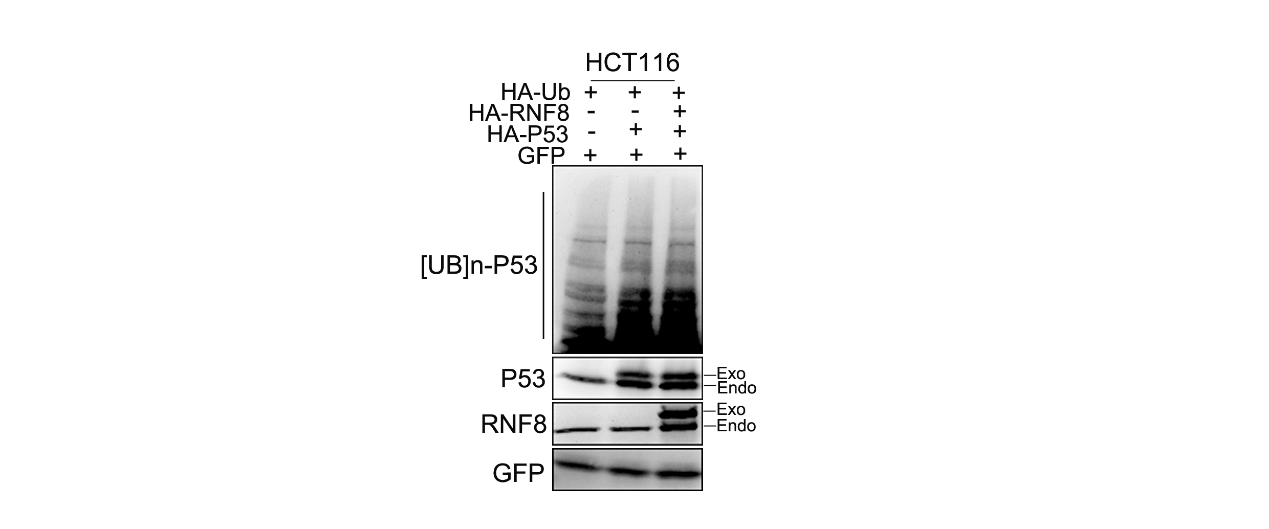
**

**Figure S7**. RNF8 indirectly regulate the pro-apoptotic function of p53. HCT116 cells were transfected with indicated plasmids for 48 h, then treated with or without 10 μM Eto. the expression of the indicated proteins was detected by immunoblot. GFP was used as the loading control.

**Figure S8**

**
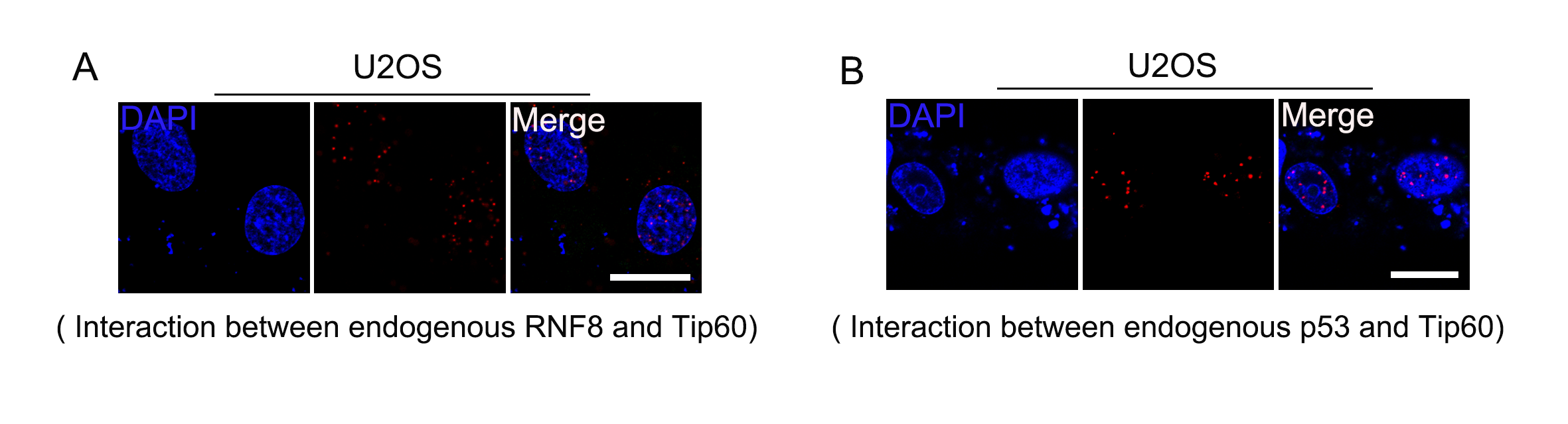
Figure S8**. Tip60 can directly interact with p53 and RNF8 respectively. A. In situ PLA using proximity probes against RNF8 and Tip60 was performed to visualize RNF8/Tip60 heterodimers in cultured human HEK-293T cells. Scale bars = 20 μm. B. In situ PLA using proximity probes against p53 and Tip60 was performed to visualize p53/Tip60 heterodimers in cultured human HEK-293T cells. Scale bars = 20 μm.

**Figure S9**

**
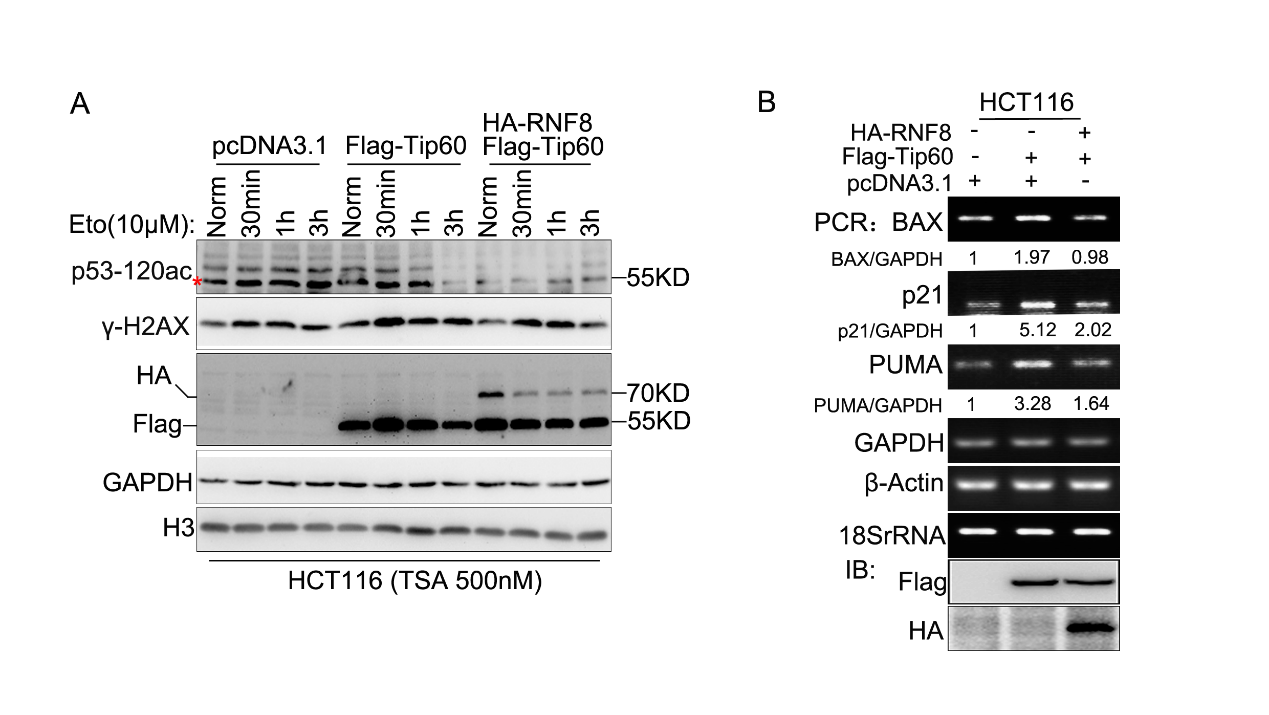
­**

**Figure S9**. RNF8 inhibits the pro-apoptotic function of p53 by regulating the functions of Tip60. (**A**) HCT116 cells were transfected with indicated plasmids for 48 h, then incubated with 10 μM Eto for 20 min and repaired for indicated times. The cells were treated with 500 nM TSA for 6 h before extracted. The expression of the indicated proteins was detected by immunoblot. GFP was used as the loading control. (**B**) HCT116 cells were transfected with indicated plasmids for 48 h, total RNA was extracted and reversely transcribed into cDNA, and the expression of related genes was detected by PCR.

**Figure S10**

**
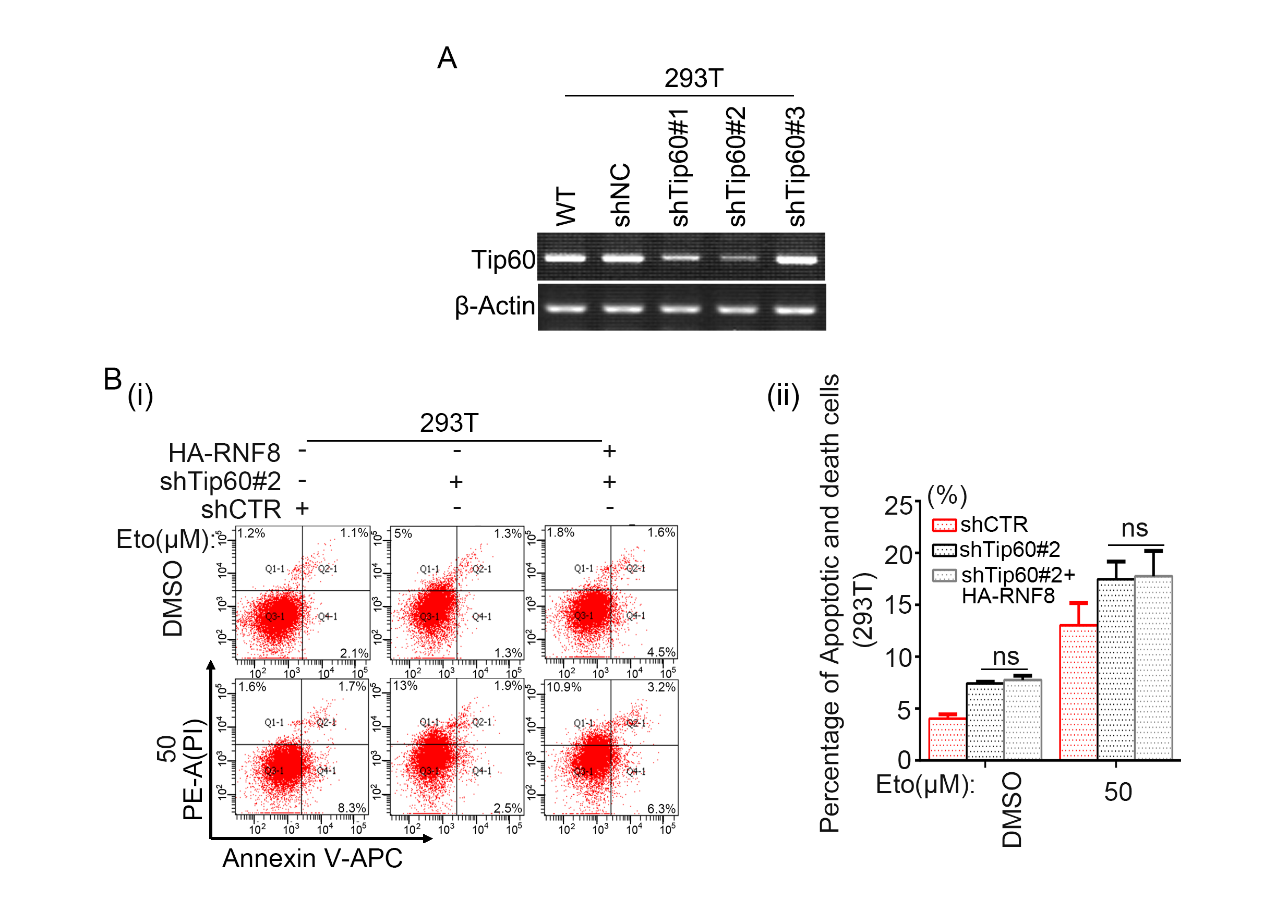
**

**Figure S10.** RNF8 did not affect the apoptosis of HEK-293T cells with low expression of Tip60. A. The interference efficiency of Tip60 in HEK-293T cells was determined by RT-PCR. B-(i). Different types of HEK-293T cells were transfected with indicated plasmids for 48 h and then incubated with 0, 50 μM of Eto for 24 h, and the flow cytometry analysis of annexin V-FITC/PI staining was conducted to examine the dead cells. (ii).The total number of PI-positive, annexin V-positive and annexin V/PI double-positive cells were quantified. ns, not significant, n≥3.

**Figure S11**

**
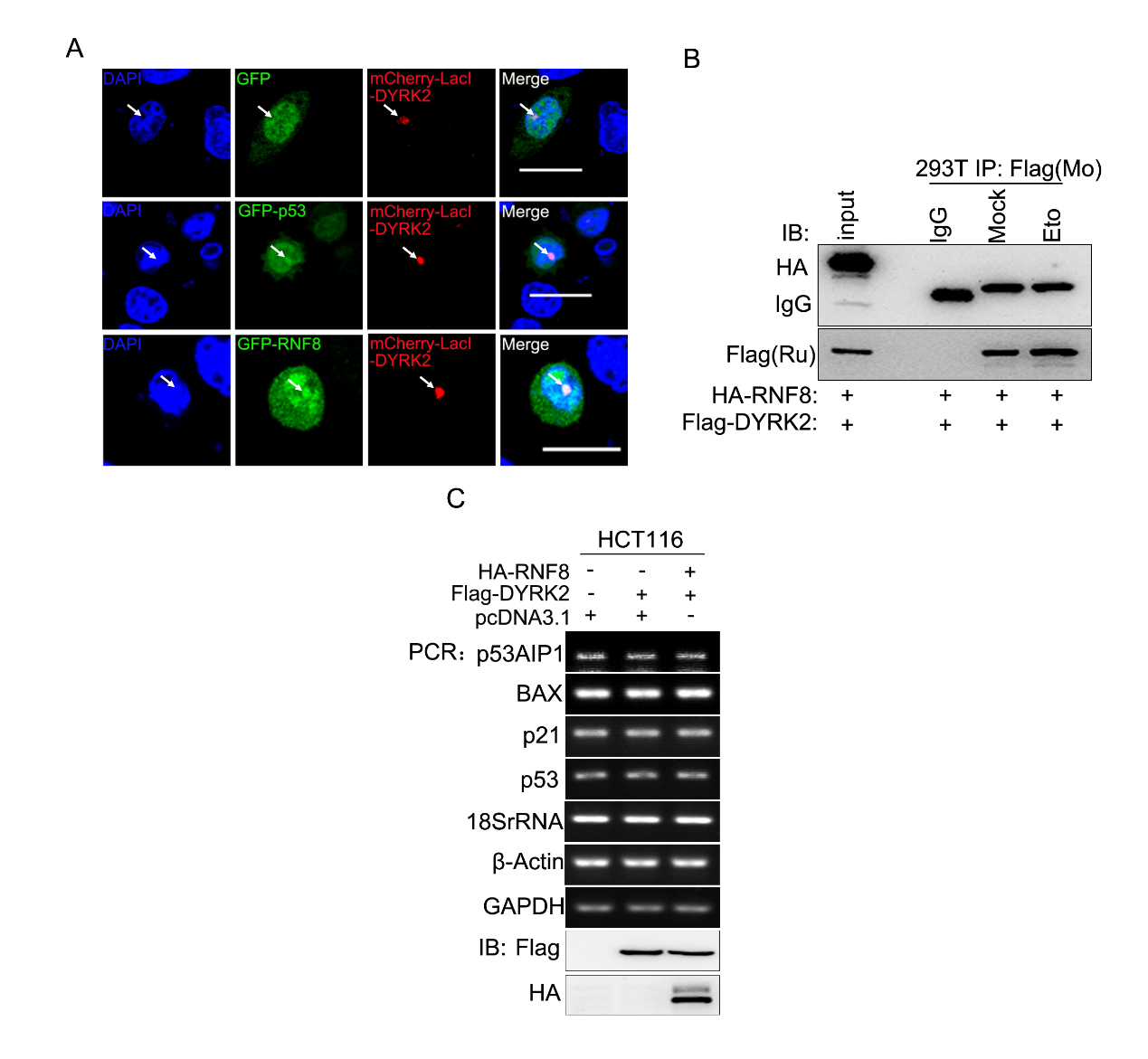
**

**Figure S11**. DYRK2 has a low effect on the regulation of RNF8 on p53 pro-apoptotic activity. (**A**) The co-localization of DYRK2 with GFP, GFP-p53 and GFP-RNF8. (**B**) Co-IP assays were performed using HEK-293T cells to check the interaction between RNF8 and DYRK2 with or without Eto treatment. Co-IP sample were resolved by SDS–PAGE and immunoblotted for the indicated antibodies. (**C**) HCT116 cells were transfected with indicated plasmids for 48 h, total RNA was extracted and reversely transcribed and the expression of related genes was detected by PCR.
